# Adsorption and anti-corrosion characteristics of vanillin Schiff bases on mild steel in 1 M HCl: experimental and theoretical study†

**DOI:** 10.1039/c9ra07982c

**Published:** 2020-03-05

**Authors:** Sanjoy Satpati, Sourav Kr. Saha, Aditya Suhasaria, Priyabrata Banerjee, Dipankar Sukul

**Affiliations:** Department of Chemistry, National Institute of Technology Durgapur West Bengal 713209 India dipankar.sukul@ch.nitdgp.ac.in +91 9434788066; Surface Engineering & Tribology Group, CSIR-Central Mechanical Engineering Research Institute Durgapur 713209 India; Academy of Scientific and Innovative Research CSIR-CMERI Campus Durgapur 713209 India pr_banerjee@cmeri.res.in +91-343-6452220; Faculty of Engineering, Hokkaido University Kita-13, Nishi-8, Kita-ku Sapporo Hokkaido 060-8628 Japan

## Abstract

Herein, two Schiff base derivatives of vanillin and divanillin with 2-picolylamine, namely, 2-methoxy-4-((pyridin-2-ylmethylimino)methyl)phenol (compound A) and 3,3′-dimethoxy-5,5′-bis-((pyridin-2-ylmethylimino)methyl)-[1,1′-biphenyl]-2,2′-diol (compound B), respectively, were synthesized. Additionally, their adsorption characteristics and corrosion inhibition behavior were compared for mild steel in 1 M HCl using electrochemical impedance spectroscopy, potentiodynamic polarization and weight loss methods. Compound B was found to impart a better anti-corrosive effect (around 95% inhibition efficiency at 313 K) than compound A. The inhibitors act as effective mixed-type inhibitors and exhibit Langmuir-type adsorption behaviour. The kinetic–thermodynamic parameters together with the data obtained from density functional theory (DFT) and molecular dynamics (MD) simulations illustrate the mechanism of corrosion and mode of adsorption of both inhibitors on the metal surface. The better corrosion mitigation propensity of the dimeric form of the inhibitor (compound B) over the monomeric form (compound A) was tested experimentally and explained according to the theoretical data.

## Abstract 2

Two Schiff base derivatives of vanillin and divanillin with 2-picolylamine are synthesized and their anti-corrosive propensity for mild steel in aqueous HCl are compared.

## Graphical abstract

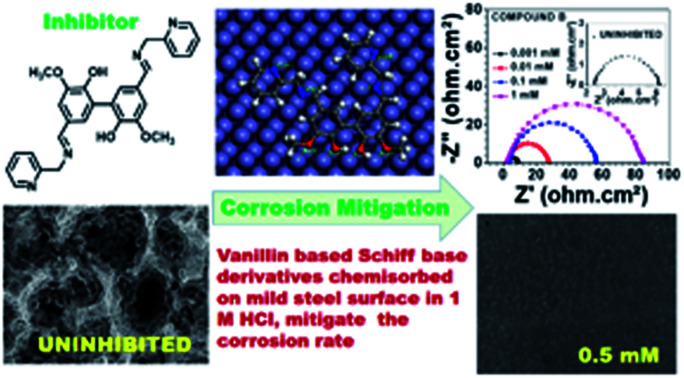

## Introduction

The application of suitable corrosion inhibitors for the control of corrosion in metals and alloys is very important.^1–4^ However, due to ecological concerns, the use of inorganic inhibitors is gradually being restricted. This has resulted in a surge of studies involving organic corrosion inhibitors. Organic compounds containing N, S, and O atoms generally show good inhibition efficiency for mild steel in acidic media.^5–12^ In addition to various heterocycles,^13^ amines^14^ and imines,^15^ different other classes of organics, such as amino acids,^16,17^ vitamins,^18^ polysaccharides,^19^ surfactants,^20^ polypeptides,^21^ lipids,^22^ polyphenols^1^ and others, have been reported to act as efficient corrosion inhibitors. Schiff bases are versatile compounds, which are synthesized *via* the condensation of primary amines and carbonyl compounds, and used widely in pharmaceuticals, agrochemicals and materials science.^23–25^ In this work, we aimed to investigate the corrosion inhibition properties of two newly synthesized Schiff base derivatives of vanillin and divanillin with 2-picolylamine for mild steel in 1 M HCl.

**Fig. 1:**
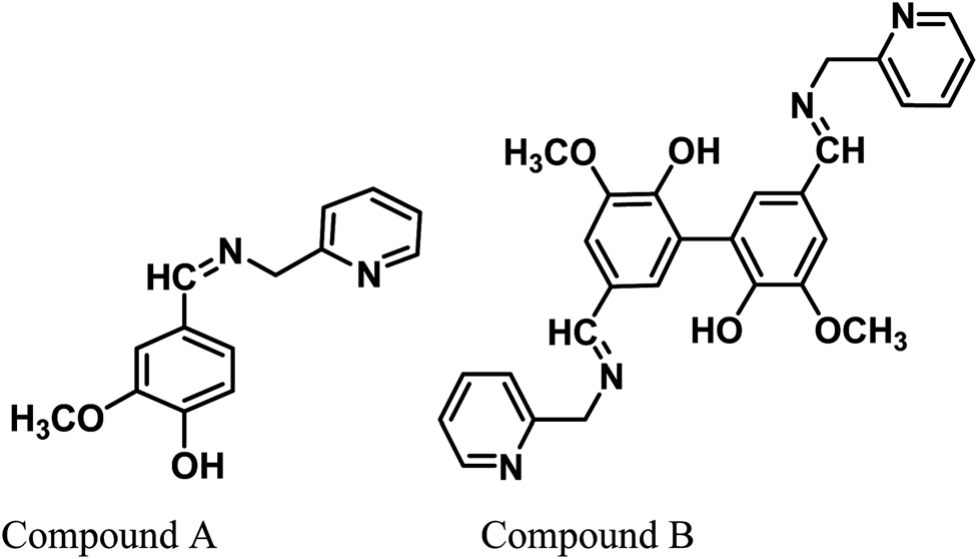
Chemical structures of the vanillin-based Schiff bases.

Vanillin, a biomass-derived phenolic aldehyde, is widely used as a flavoring agent in foods, beverages and pharmaceuticals owing to its anti-microbial and anti-oxidant properties.^26^ It was first extracted from vanilla beans, which are primarily obtained from the orchid *Vanilla planifolia*. The synthetic production of vanillin from the abundant lignin *via* metal-catalyzed air oxidation converts it into a potential renewable feedstock chemical.^27,28^ Herein, we provide further value to vanillin and explore its potential for applications in a new arena of green corrosion inhibitors, which are essentially of bio-origin and less toxic to the environment. To date, vanillin has been tested for its anti-corrosive propensity for aluminum in acid solutions.^29^ However, since vanillin itself failed in this effort for ferrous metal, it was derivatized into a Schiff base, *i.e.*, 2-methoxy-4-((pyridin-2-ylmethylimino)methyl)phenol (compound A). Further, we synthesized divanillin, which was subsequently converted to another Schiff base, 3,3′-dimethoxy-5,5′-bis(((pyridin-2-ylmethyl)imino)methyl)-[1,1′-biphenyl]-2,2′-diol (compound B). The molecular formulae of these two Schiff bases are shown in Fig. 1. Compound B is essentially the dimeric form of compound A. One of our main intentions of this work is to compare the corrosion inhibition properties of the dimeric and monomeric forms of the vanillin Schiff bases and explain the phenomena from a theoretical viewpoint. Electrochemical impedance spectroscopy, potentiodynamic polarisation and the weight loss method were used to study the anticorrosive behavior of these Schiff bases for mild steel in an HCl medium. The corresponding corrosion inhibition efficiency and thermodynamic and activation parameters involved in the process in the presence and absence of inhibitors were calculated. Also, a detailed theoretical study using DFT and molecular dynamics (MD) simulations was performed to determine the quantum chemical parameters and to establish the experimentally obtained results.

## Experimental

### Synthesis of compound A

0.761 g vanillin (5 mmol) was dissolved in 40 mL of methanol followed by the dropwise addition of 0.541 g of 2-(aminomethyl)pyridine (5 mmol). The mixture was then allowed to reflux for 15 h. The pale yellow colour product obtained after evaporation of the solvent was purified by solvent extraction using chloroform and water. The resulting product after the evaporation of chloroform was washed with diethyl ether to remove any unreacted vanillin. Finally, the product was dried and used for further study (yield 70%).

### Synthesis of divanillin

Divanillin, the symmetrical dimer of vanillin, was synthesized from vanillin using FeCl_3_.^30^ 2.973 g (11 mmol) FeCl_3_·6H_2_O was dissolved in 50 mL water in a round-bottom flask equipped with a magnetic stirrer, oil bath and condenser. To this solution, 1.521 g of vanillin was added and suspended under constant stirring. The mixture was heated to 50 °C under constant stirring for 6 h. The mixture was then cooled in an ice bath and the resultant precipitate was filtered and washed with water and methanol, and then finally dried to obtain an ash coloured product (yield 55%).

### Synthesis of compound B

1.511 g divanillin (5 mmol) was added to a mixture of 70 mL of methanol and 10 mL DMF and stirred under heating to make divanillin soluble in the medium. This was followed by the dropwise addition of 1.082 g of 2-(aminomethyl)pyridine (10 mmol). The mixture was then allowed to reflux for 18 h. A greenish solid precipitate appeared after cooling the reaction mixture. The product was collected by filtration and washed with methanol and then diethyl ether several times and finally dried (yield 70%).

Compound A, divanillin and compound B were characterized *via*^1^H NMR (Fig. S1–S3 in the ESI,†), FTIR (Fig. S4 in ESI†), and ESI mass spectroscopy for compounds A and B (Fig. S5 and S6 in ESI†) and EI mass spectroscopy for divanillin (Fig. S7 in ESI†).

### ^1^H NMR of compound A

*δ* ppm (DMSO-d6, 400 MHz): *δ* ppm (DMSO-d6, 400 MHz): 3.734 ppm (s, 3H, H atoms of –OCH_3_ group), 4.737 ppm (s, 2H, H atoms of methylene group attached to pyridine ring), 6.777 to 6.797 ppm (d, 1H, H atom of benzene ring nearest to –OH group), 6.935 to 6.964 ppm (d, 1H, δ-H atom of pyridine ring), 7.132 to 7.159 ppm (t, 1H, β-H of pyridine ring), 7.206 to 7.225 (d, 1H, 1H, H atom of benzene ring opposite to –OCH_3_ group), 7.333 ppm (s, 1H, H atom of benzene ring nearest to –OCH_3_ group), 7.707–7.731 ppm (t, 1H, γ-H of pyridine ring), 8.313 to 8.322 ppm (d, 1H, α-H of pyridine ring), 8.460 ppm (s, 1H, H atom attached to C atom of imine bond).

### ^1^H NMR of divanillin

*δ* ppm (DMSO-d6, 400 MHz): 3.867 ppm (s, 6H, H atoms of –OCH_3_ group), 7.367 ppm (s, 4H, H-atoms of aromatic rings), 9.775 ppm (s, 2H, H-atoms of aldehyde group).^31,32^

### ^1^H NMR of compound B

*δ* ppm (DMSO-d6, 400 MHz): 3.717 ppm (s, 6H, H atoms of –OCH_3_ group), 4.764 ppm (s, 4H, H atoms of two methylene group attached to two pyridine ring), 6.921 to 6.950 ppm (d, 2H, δ-H atom of pyridine ring), centered at 7.225 ppm (4H, H atom of benzene ring of divanillin unit), 7.409 to 7.454 ppm (t, 2H, β-H of pyridine ring), 7.796 to 7.845 (t, 2H, γ-H of pyridine ring), 8.349 to 8.340 ppm (d, 2H, α-H of pyridine ring), 8.492 ppm (s, 2H, H atom attached to C atom of imine bond).

### FTIR spectrum of divanillin

Divanillin consists of a benzene rings together with aldehyde groups, phenolic –OH groups and ether (–OCH_3_) groups. The broad absorption peak at around 3261 cm^−1^ indicates the presence of –OH groups (O–H stretching). The weak peak around 3050 cm^−1^ is due to the presence of aromatic C–H stretching. Another weak band at around 2969 cm^−1^ and 2942 cm^−1^ is due to the C–H stretching of the –OCH_3_ group. The strong and sharp peak at 1675 cm^−1^ is due to the C–O stretching vibration of the aldehyde group attached to aromatic rings.^32,33^ The sharp and strong peaks in the region of 1587 cm^−1^ to 1509 cm^−1^ are attributed to the presence of aromatic C=C. The peaks in the region of 1456 cm^−1^ to 1400 cm^−1^ are due to the in-plane C–H bending vibrations. The appearance of the peak at 1353 cm^−1^ is due to the C–C stretching. The peak at 1281 cm^−1^ corresponds to the C–O bending and the peaks at 1259 and 1192 cm^−1^ the C–O stretching vibrations.^33^ The other peaks are due to the different modes of the C–H, C–C, O–H, C–O bending vibrations.^33^

### FTIR spectra of compounds A and B

In the vanillin moiety of compound A and compound B, the aldehyde group is converted into an imine group and a new pyridine ring is introduced. Thus, the peak for the aldehyde is absent and new peaks appear at 1639 cm^−1^ for compound A and 1636 cm^−1^ for compound B due to the C=N (imine bond) stretching. The broad peak centered at ∼3415 cm^−1^ indicates the presence of –OH groups (O–H stretching). The appearance of peaks in the region of 1595 cm^−1^ to 1435 cm^−1^ is attributed to the presence of both benzene and pyridine aromatic rings. The other peaks are due to the different modes of the C–H, C–C, O–H, and C–O stretching and bending vibrations.

### ESI-mass spectra of compounds A and B

The appearance of a prominent molecular ion peak at *m*/*z* 243.2 [L + H^+^] confirms the formation of compound A. Due to the very low solubility of compound B in common organic solvent, the mass spectrum of compound B is not very clear and contains noise. However, the presence of a molecular ion peak at *m*/*z* at 483.1264 (∼50%) confirms the formation of compound B. The presence of peaks at *m*/*z* 451.127 (L-CH_3_OH + H), 405.1625 (L-py unit + H), 328.1636 (L-2py unit + H), 314.1520 (L-py unit − py-CH_2_ unit + H), 511.1565 (2L + 3H_2_O + 2K), and 600.2269 (L + DMSO + K) also confirms the formation of compound B.

### EI-mass spectrum of divanillin

The appearance of a very strong and sharp peak at 302 in the EI mass spectrum confirms the formation of divanillin.

### Specimen and solution

Cylindrical-shaped sample specimens were cut from commercially available mild steel rods with a diameter of 1 inch having the following composition: 0.22 C, 0.31 Si, 0.60 Mn, 0.04 P, 0.06 S and the remainder iron (wt% composition). A shiny metal surface was prepared by rubbing the cut sample pieces with different grade silicon carbide paper (from 60 to 1200), and thereafter washing thoroughly with soap, tap water, distilled water and finally with acetone, and drying in a vacuum desiccator. All the reagents used were AR grade. Vanillin was obtained from Merck India, 2-picolylamine from Sigma India and HCl (1 M) solution was prepared using 37% HCl from Merck India.

### Electrochemical measurements

All electrochemical experiments, *i.e.* electrochemical impedance spectroscopy (EIS) and Tafel extrapolation method were performed using a conventional 3-electrode system (model: Gill AC, ACM Instruments, UK). A mild steel sample with an exposed surface area of 1 cm^2^ functioned as the working electrode, a Pt mesh electrode as the auxiliary electrode and saturated calomel electrode (SCE) as the reference electrode (RE). Before the measurements, the working electrode in the cell was kept in contact with 350 mL test solution for 35 min to achieve the steady state condition.

Potentiodynamic polarization measurements were performed at a scan rate of 0.5 mV s^−1^ with in the potential range of ±250 mV from the respective rest potential values. Inhibition efficiency, *η*_P_ (%), is defined as:1
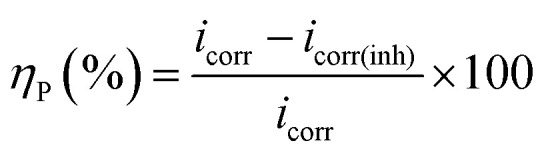
where, *i*_corr_ and *i*_corr(inh)_ are the corrosion current density of the uninhibited and inhibited specimens, respectively, which were estimated using the Tafel extrapolation method.

EIS measurement was performed within the frequency range of 0.1 MHz to 10 MHz with an AC amplitude of ±10 mV (rms) at the rest potential. The inhibition efficiency of the inhibitor is defined as follows:2
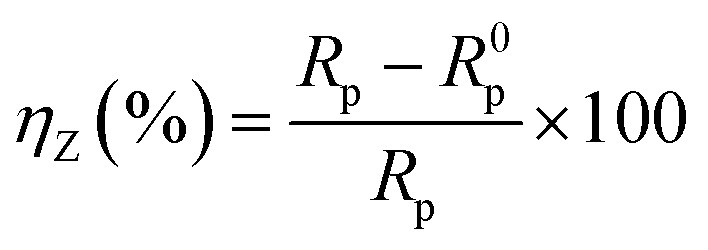
where *R*_p_ and *R*^0^_p_ represent the impedance value in the presence and absence of the inhibitor, respectively. *R*_p_ accounts for a combined effect of different resistances operating at the metal-electrolyte interface, such as the charge transfer resistance and resistance due to adsorbed corrosion products as well as inhibitors.

### Weight loss measurements

The effect of temperature on the corrosion inhibition efficiency, rate of corrosion of the inhibited and uninhibited specimens, different thermodynamics and activation parameters related with the corrosion inhibition process were evaluated using the following method. Mild steel coupons (wt% composition: 0.19 C, 0.21 Si, 0.21 Mn, 0.01 P, 0.01 S and the remainder iron) with the dimensions of 2.5 cm × 2.5 cm × 0.1 cm were treated properly, as previously described (in specimen and solution section). After taking the initial weight, the coupons were immersed in 350 mL of aqueous HCl (1 M) in the absence and presence of inhibitors. After 6 h of exposure, the coupons were taken out, and washed under running water using a bristled brush. Coupons were then washed with distilled water and acetone and dried in a vacuum desiccator before taking their final weight. During the experiment with prolonged exposure, the volume of the solution was maintained by adding the requisite amount of distilled water when required. The percentage inhibition efficiency, *η*_W_ (%), was calculated using following the relation:3
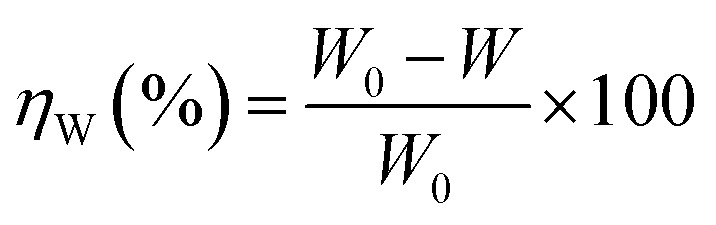
where *W*_0_ and *W* are the weight loss of the metal samples in acid medium in the presence or absence of the inhibitor.

Since organic inhibitors are mostly adsorption-type inhibitors, a direct correlation between the degree of surface coverage (*θ*) with inhibition efficiency over the whole concentration range is generally assumed. Following this assumption, *θ* was calculated using the *η*_W_ (%) values according to the following equation:

4 *θ* = *η*_W_ (%)/100

### Surface morphology

The morphological study was accomplished by keeping the mild steel samples in 1 M HCl medium in the presence and absence of inhibitors for 6 h, and thereafter the sample specimen was gently washed with acetone and distilled water and dried. The surface morphology was observed using scanning electron microscopy (Hitachi, S-3000N).

### Computational details for quantum chemical calculation

The quantum chemical parameters for the inhibitor molecules were calculated *via* the density functional theory (DFT) using the ORCA program (version 2.7.0). Geometrical optimisation of the inhibitors was performed at the B3LYP^34^ functional level using the triple-ζ quality basis set TZV (P) with one set of polarization functions on the N, O and S atoms. For atoms such carbon and hydrogen, we used slightly smaller polarized split-valence SV (P) basis sets, which are double-ζ quality in the valence region and have a polarizing set of d functions on atoms other than hydrogen.^1,34^ SCF calculations were converged tightly (1 × 10^−8^ Eh in energy, 1 × 10^−7^ Eh in density and 1 × 10^−7^ in maximum element of the DIIS error vector). All theoretical calculations were performed in the aqueous phase because electrochemical corrosion occurs in the aqueous phase considering the solvent as a continuum of uniform dielectric constant (*ε*), where the solute is placed as a uniform series of interlocking atomic spheres.^35^ Various intrinsic molecular parameters such as electron affinity (*A*), ionization potential (*I*), electronegativity (*χ*, indicates the ability of a group of atoms to attract electrons towards itself), global hardness (*η*, measure of the resistance of an atom towards charge transfer) and global softness (*σ*, susceptibility of inhibitor molecules towards charge transfer) were calculated using the following equations^36,37^

5 *χ* = (*I* + *A*)/2

6 *η* = (*I* − *A*)/2

7 *σ* = 1/*η* = 2/(*I* − *A*)

8 *I* = −*E*_HOMO_

9 *A* = −*E*_LUMO_

The fraction of electrons transferred from the inhibitor molecule to the metallic atom (Δ*N*) was calculated using the following relation:^36^10
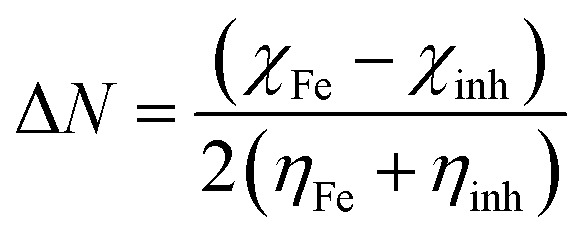


The concept of Δ*N* is based on the assumption that between two interacting systems of different electronegativities (here, the metallic surface and an inhibitor molecule), the electron will flow from the molecule of lower electronegativity to that with a higher value until their chemical potentials become the same. To evaluate the value of Δ*N*, the electronegativity of Fe (*χ*_Fe_) was replaced by the work function of the Fe (1 1 0) surface (most stable densely packed surface among the Fe surfaces), *ϕ*_Fe_ = 4.82 eV (ref. 38 and 39) and the global hardness of iron, *η*_Fe_, was taken as zero considering *I* = *A* for the metallic bulk.

### Local reactivity analysis (Fukui indices)

The local reactivity of the molecules was analysed through evaluation of the Fukui indices using the Dmol^3^ module in Material Studio™ version 6.1 by Accelrys Inc, San Diego, CA. All calculations were performed by applying the B3LYP exchange–correlation function and the double numeric with polarization (DNP) basis set. Here, the Fukui functions were obtained through the finite difference approximation using Hirschfield population analysis (HPA).^40–42^ By applying the concept HSAB principle^43^ in a local manner, it can be interpreted that the regions of a molecule where the Fukui function is large are chemically softer than the regions where the Fukui function is small. The Fukui function at a point, *r*, in the space around the molecule is defined as the first order derivative of electron density at that point with respect to the number of electrons, *N*, present in the molecule at a constant external potential, *v*.^40–42^11
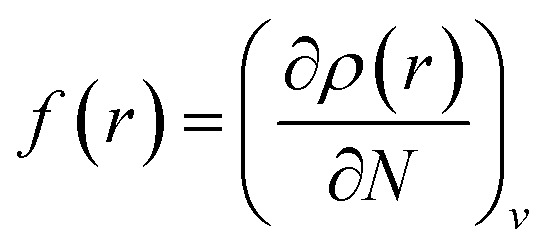


Since the electron density is discontinuous with respect to the number of electrons, left hand and right hand side derivatives were introduced as:12
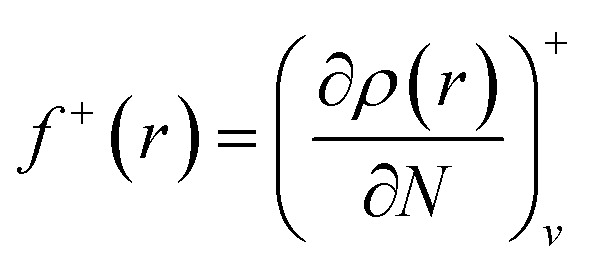
13
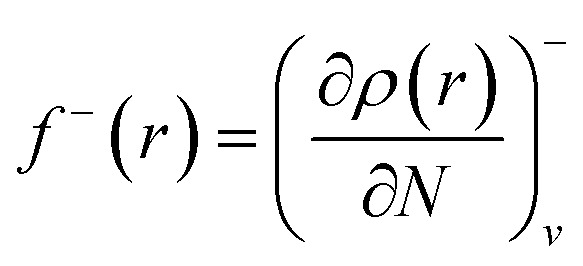
where *f*^+^ can be used to probe the reactivity when electrons are added to the system (attack of a nucleophile) and *f*^−^ to probe the reactivity when electrons are extracted from the system (attack of an electrophile). Taking the finite different approximation, a condensed form of these functions is proposed as:^44^

14 *f*^+^_k_ ≈ *q*_k_(*N* + 1) − *q*_k_(*N*) (for nucleophilic attack)

15 *f*^−^_k_ ≈ *q*_k_(*N*) − *q*_k_(*N* − 1) (for electrophilic attack)

where *q*_k_(*N* + 1), *q*_k_(*N*) and *q*_k_(*N* − 1) are the gross atomic population (*i.e.* gross atomic charge) of the atom k in the *N* + 1, *N* and *N* − 1 electron systems, respectively.

### Molecular dynamics simulation

To calculate the interaction energies between the Fe (1 1 0) surface and our inhibitor molecules, we used the molecular dynamics (MD) simulation technique. Accordingly, we used the Material Studio™ software 6.1 (from Accelrys Inc.). In this simulation process, the interaction between the studied molecules and iron surface was carried out in a simulation box of (40.11 Å × 40.11 Å × 78.00 Å) with periodic boundary conditions to avoid any arbitrary boundary effect. Here, we used ten layers of iron atoms to provide sufficient depth to overcome the issue related to the cut-off radius. In this investigation, a three-layered simulation box was created. The first layer contained an Fe slab and the second layer was the solution slab, which contained H_2_O (150), together with H_3_O^+^ and Cl^−^ ions (15 each) as well as the inhibitor molecule, and the remaining part of the box was the vacuum layer. The presence of H_3_O^+^ and Cl^−^ ions makes the MD simulation closer to the real system. After construction of the simulation box, MD simulation was carried out using the COMPASS (Condensed Phase-optimized Molecular Potentials for Atomistic Simulation Studies) *ab initio* force field. In general, the parameterization procedure was divided into two phases: (i) *ab initio* parameterization and (ii) empirical optimization. The MD simulation was performed at 298.0 K using a canonical ensemble (NVT) with a time step of 1.0 fs and for a simulation time of 100 ps. All the bulk atoms in the Fe (1 1 0) surface were kept frozen and all the concerned molecules were allowed to interact with the metal surface freely during the entire simulation process. The interaction energy (*E*_interaction_) between the inhibitor molecule and the Fe (1 1 0) surface was calculated using the following equation:^1,9,10^

16 *E*_interaction_ = *E*_total_ − (*E*_surface+H_2_O+H_3_O^+^+Cl^−^_ + *E*_inhibitor_)

where the total energy of the simulation system is defined as *E*_total_, the energy of the iron surface together with H_2_O, H_3_O^+^, Cl^−^ is classified as *E*_surface+ H_2_O+H_3_O^+^+Cl^−^_ and that of the free inhibitor molecule as *E*_inhibitor_.

## Results and discussion

### Polarization measurements

**Fig. 2:**
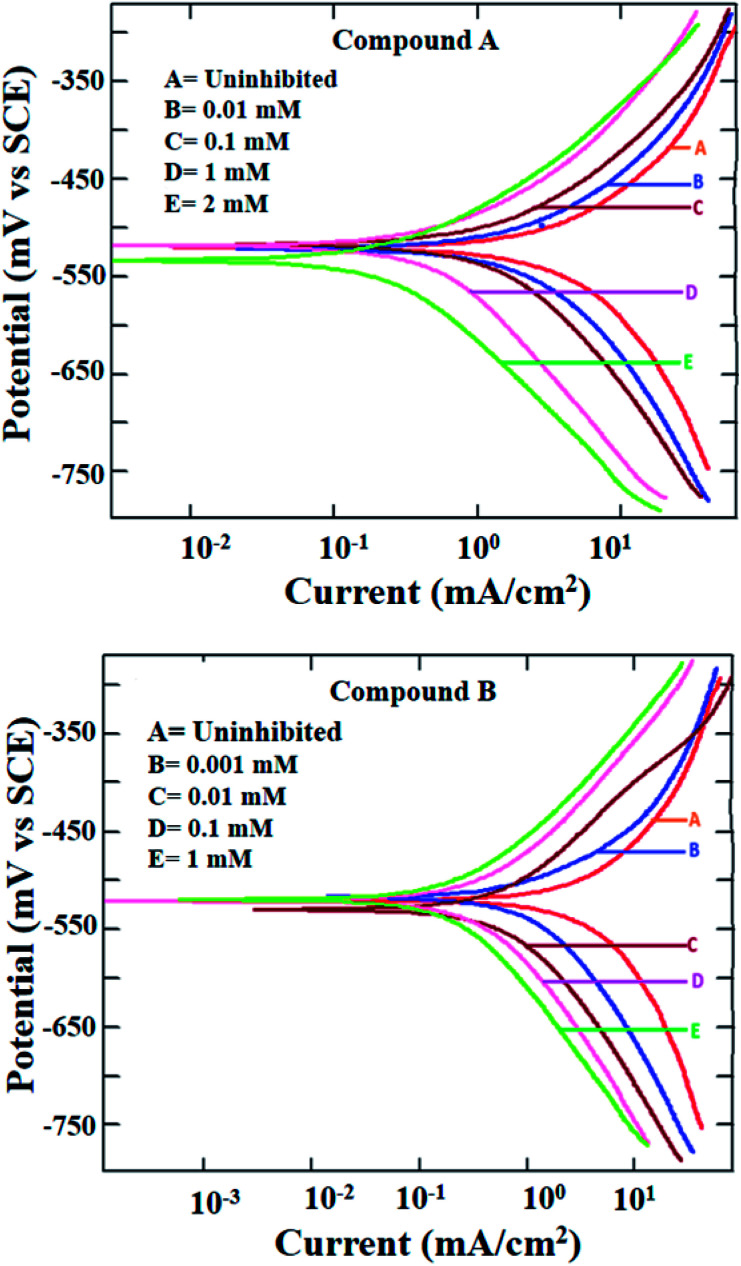
Potentiodynamic polarization plots for mild steel in 1 M HCl in the presence of compound A (top) and compound B (bottom) at 303 K.

The potentiodynamic polarization measurement was performed with a range of concentrations for both inhibitor molecules and at two different temperatures of 303 and 323 K. The polarization plots for mild steel in 1 M HCl at 303 K in the presence of compounds A and B are shown in Fig. 2. Various corrosion parameters such as the corrosion potential (*E*_corr_), corrosion current density (*i*_corr_) and anodic and cathodic Tafel slopes (*b*_a_ and *b*_c_, respectively) were derived following the Tafel extrapolation method (representative examples shown in Fig. S8 in the ESI†), and tabulated in Table 1. The polarization plots and corrosion parameters correspond to the typical mixed-type corrosion inhibitor behavior for both compounds.^45^ Both the cathodic and anodic currents diminished systematically with an increase in the concentration of the inhibitors, without much alteration in the cathodic and anodic slopes. The corrosion potentials also remained within a narrow range of ±10 mV.

**Table 1:** Potentiodynamic polarisation data for mild steel in 1 M HCl in the presence and absence of compounds A and B at 303 K

Conc. (mM)	−*E*_corr_ (mV/SCE)	*b*_a_ (mV dec^−1^)	−*b*_c_ (mV dec^−1^)	*i*_corr_ (μA cm^−2^)	*η*_P_, %
Uninhibited	521	121.4	158.7	3254	—

**With compound**A					
0.001	514	103.8	129.5	2257	22.3
0.010	518	104.2	130.7	1681	48.3
0.10	518	103.4	129.5	1140	64.9
0.25	513	106.3	131.2	709	78.2
0.50	529	102.9	132.6	675	79.3
1.0	517	101.1	128.2	479	85.3
2.0	532	100.6	126.3	260	92.0

**With compound**B					
0.001	518	102.9	121.6	1435	55.9
0.010	529	102.3	122.5	545	83.2
0.10	520	97.1	119.9	296	90.9
0.25	523	100.3	119.8	280	91.4
0.50	520	99.2	120.1	249	92.3
1.0	520	99.5	121.4	194.8	94.0

Mixed-type inhibition by any inhibitor indicates the retardation of both the cathodic hydrogen evolution reaction and anodic metal oxidation reaction at the cathodic and anodic reaction sites, respectively. When the inhibitor is capable of accepting electrons from the metal surface, a deficiency in electrons results in the inhibition of the rate of reduction of hydronium ions producing hydrogen gas. Similarly, a reduced rate of metal oxidation reaction is indicative of the fact that the inhibitors are prone to donate electrons at the anodic sites of the metal surface, thereby increasing the electron density at the metal-electrolyte interface. Thus, it can be concluded that both inhibitors are capable of donating electrons at the anodic reaction sites and accepting electrons at the cathodic reaction sites.

Due to the extremely low solubility of divanillin in 1 M HCl, its electrochemical study could not be carried out. The other two reactants, *i.e.*, vanillin and 2-(aminomethyl)pyridine did not exhibit corrosion protection to any significant extent (inhibition efficiency being 60–70%, Fig. S9 and Table S1 in ESI†). Thus, the enhancement of corrosion protection of mild steel in aqueous HCl was evident when vanillin and 2-(aminomethyl)pyridine reacted and produced the Schiff base, compound A. Accordingly, the resultant imine group present in compound A must play a role in providing this significantly greater effect. The other possible molecular parameters will be considered following the quantum molecular result.

Further, at the elevated temperature of 323 K, the rate of corrosion was higher in the presence of both compounds A and B, which is common for any activation energy-controlled reaction. However, the inhibition efficiencies at this higher temperature for both compounds showed almost the same value as that at 303 K (Fig. S10 and Table S2 in ESI†). Thus, these compounds can be applied as effective mixed-type corrosion inhibitors, at least up to 323 K.

### Electrochemical impedance measurements

**Fig. 3:**
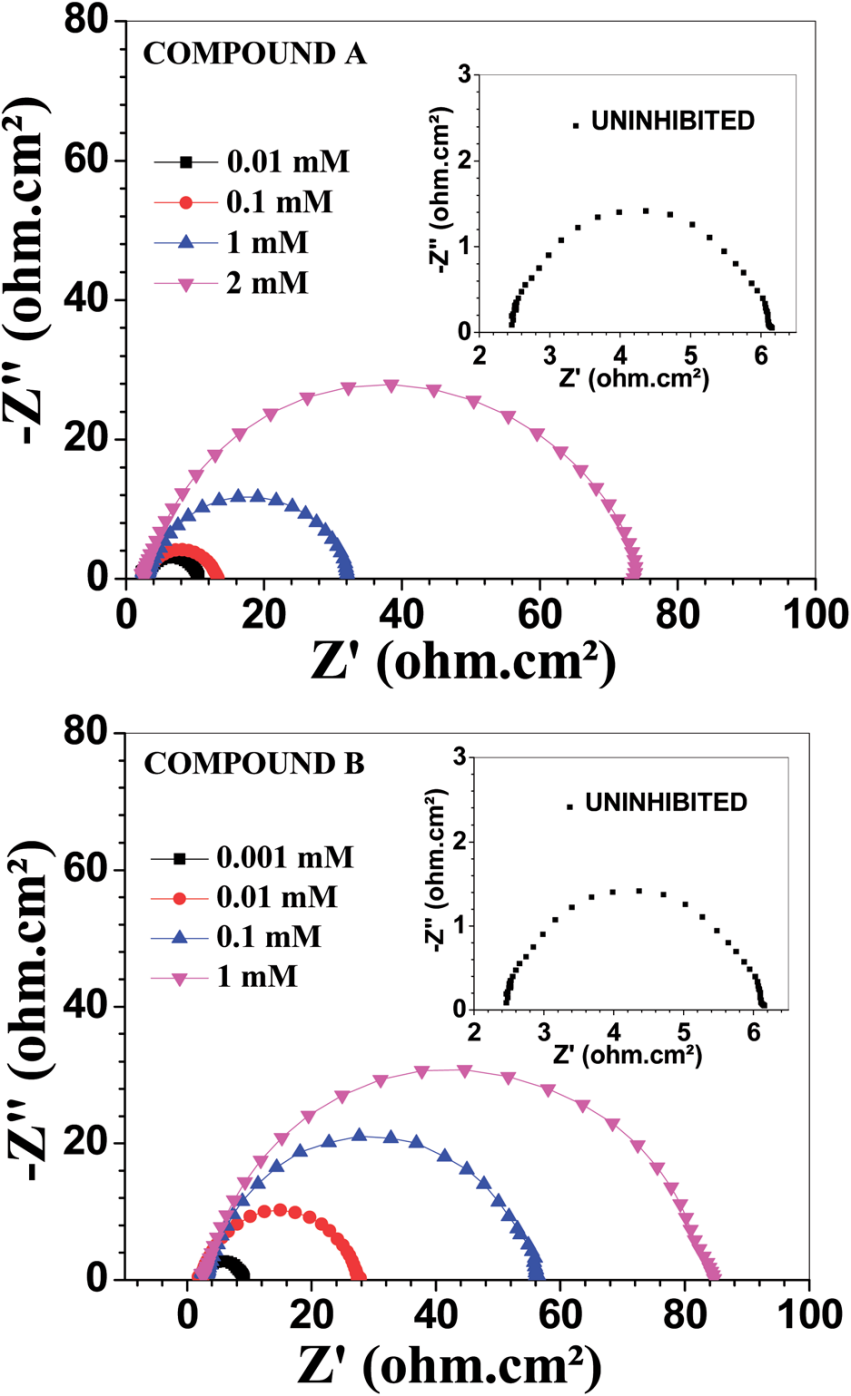
Nyquist plots for mild steel in 1 M HCl in the presence of compound A (top) and compound B (bottom) at 303 K.

**Fig. 4:**
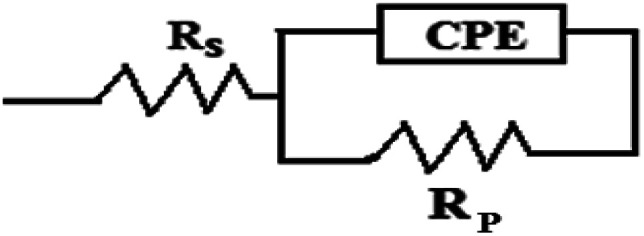
Equivalent circuit model used to fit the impedance spectra.

Fig. 3 depicts the Nyquist plots of mild steel in 1 M HCl with different concentrations of compounds A and B together with the uninhibited specimen at 303 K. The corresponding impedance and phase angle plots (Bode plots) are shown in ESI (Fig. S11 and S12†). These plots clearly reveal that an increase in inhibitor concentration (both compounds A and B) leads to a concomitant enhancement in the diameter of the capacitance loops. Consequently, this indicates an increase in polarization resistance (*R*_p_). *R*_p_ is a collective parameter comprising of various resistive factors operating at the metal-electrolyte interface. These factors include charge transfer resistance (*R*_ct_) for both metal ion oxidation and hydrogen ion reduction, resistance of the adsorbed organic inhibitor layer and resistance of the adsorbed corrosion products. All these parameters are interrelated and cannot be factorized individually.^46,47^ Overall, with a gradual increase in inhibitor concentration, the degree of surface coverage and thickness of the adsorbed inhibitor layer increase, replacing the pre-adsorbed water molecules and ions present in the electrolyte solution, and thus increasing the *R*_p_ value.^46–48^ The non-perfect semi-circle nature of the Nyquist plots can be explained in terms of surface heterogeneity due to the formation of an adsorbed layer of the inhibitors together with the corrosion product.^48^ The Nyquist plots in the absence and presence of inhibitors reveal the existence of one capacitive loop, and the absence of an inductive loop. The corresponding Bode phase angle plots contain one maximum without any hump, while the Bode impedance plots show only one negative fluctuation. All these phenomena correspond to the involvement of only one time constant. Accordingly, the observed Nyquist plots were fitted with an equivalent circuit model, as shown in Fig. 4, where *R*_p_ stands for the polarization resistance and *R*_s_ stands for the solution resistance. The constant phase element (CPE) was used in the equivalent circuit instead of the double-layer capacity (*C*_dl_) to accommodate the observed depressed semi-circle nature in the Nyquist plots.^49^ Accordingly, the chi-square value in the order of 10^−4^ confirms the validity of the model and goodness of the fit. The fitted parameters together with the inhibition efficiency is presented in Table 2.

**Table 2:** EIS data for mild steel in 1 M HCl in the presence and absence of compounds A and B at 303 K

Conc. (mM)	*R*_p_ (Ω cm^2^)	*Q* (μΩ^−1^ s^*n*^ cm^−2^)	*n*	*C*_dl_ (μF cm^−2^)	*η*_z_, %	*χ* ^2^ × 10^4^
Uninhibited	3.8	1010	0.81	273.9		4.21

**With compound**A						
0.001	4.8	738	0.82	213.8	20.8	1.62
0.010	7.8	553	0.84	196.0	51.3	2.82
0.10	10.6	353	0.85	131.7	64.2	1.65
0.25	14.8	276	0.85	104.5	74.3	0.7
0.50	22.6	229	0.85	90.45	83.2	2.97
1.0	29.6	190	0.85	76.1	87.2	3.36
2.0	72.4	147	0.86	70.2	94.8	1.22

**With compound**B						
0.001	7.1	400	0.82	110.4	46.5	0.6
0.010	25.6	207	0.84	76.3	85.2	1.37
0.10	53.5	169	0.85	73.6	92.9	3.43
0.25	63.0	146	0.85	63.8	93.9	2.62
0.50	71.2	127	0.86	59.0	94.7	3.05
1.0	80.9	125	0.86	59.2	95.3	2.17

The relation between CPE and impedance is given as

17 *Z*_CPE_ = *Q*^−1^(iω)^−*n*^

where *Q* is a proportionality coefficient, *ω* is the angular frequency, *n* is a measure of surface irregularity. CPE is converted into the classical lumped elements capacitor (*C*), resistance (*R*), and inductance (*L*) when *n* = 1, 0, and −1, respectively.

The relation between polarization resistance (*R*_p_) and double layer capacitance (*C*_dl_), is given by the relation.^50,51^

18 *C*_dl_ = (*QR*_p_^1−*n*^)^1/*n*^

Table 2 demonstrates that the capacitance (*C*_dl_) values decreased gradually with an increase in inhibitor concentration. The rate of the decrease in capacitance was greater for compound B. The concept of electrochemical double-layer capacitance was first introduced by Helmholtz, and then elaborated by Stern and Geary. Different parameters such as the electrolyte dielectric constant (*ε*), surface area accessible to ions (*A*) and distance between the ion and the charged electrode surface (*d*) determine the capacitance, *C*_dl_, according to the following equation:

19 *C*_dl_ = *ε*_0_*εA*/*d*

where, *ε*_0_ is the vacuum permittivity.^52,53^ An increase in inhibitor concentration leads to a higher degree of surface coverage, and thereby reduces the effective value of *A*. Further, organic inhibitor molecules are adsorbed on the metal surface, replacing pre-adsorbed ions and water molecules. This reduces the dielectric constant of the solution adjacent to the metal electrode. Since the pre-adsorbed ions forming the double layer diffuse away from the metal surface, the effective thickness of the double layer increases.^54,55^ All these factors act cooperatively in reducing the electrochemical double layer capacitance per unit surface area in the presence of an organic corrosion inhibitor. The various corrosion rate-reducing effects were more dominant for compound B, making it the better inhibitor. This is also evident from the higher *R*_p_ and lower *C*_dl_ values for compound B compared to that at the same concentration for compound A.

The EIS data for vanillin and 2-picolyl amine is presented in the ESI (Fig. S13 and Table S3 in ESI†), which again confirms that compounds A and B have much better corrosion resistivity for mild steel than their precursors. With an increase in temperature, the *R*_p_ value of the both uninhibited and inhibited systems decreased. However, this did not affect their inhibition efficiencies. Thus, despite the lower degree of protection offered, these vanillin derivatives stand out as good corrosion inhibitors with high inhibition efficiencies at elevated temperature (Fig. S14 and Table S4 in ESI†).

### Weight loss measurement

The results obtained from the electrochemical methods were verified using the weight loss method. We tested our inhibitors at four different temperatures within the range of 293 K to 323 K with a variable inhibitor concentration after 6 h of exposure of the test specimens in HCl solution. The importance of the weight loss method to determine the rate of corrosion comes from the fact it encompasses different types of corrosion, including overall corrosion, galvanic corrosion and all possible forms of localized corrosion. The results shown in Table 3 support the conclusion derived from the electrochemical methods. Compound B exhibited better corrosion resistivity than compound A. The maximum inhibitory effect was observed at 313 K, which decreased. Regarding the effect of exposure time on the inhibition efficiency, compound B demonstrated more than 90% inhibition efficiency with a concentration of 1 mM, even after the mild steel samples were exposed to 1 M HCl for 72 h (Table S5 in the ESI†). Thus, according to all these observations, it can be concluded that the synthesized inhibitors are useful in retarding the rate of corrosion of mild steel in 1 M HCl with a high degree of effectiveness even in an elevated temperature range and high exposure time. This indicates the strong adsorptive interaction between the inhibitor molecules and the metal surface, leading to a high value of fraction of surface covered (*θ*, Table 3).

**Table 3:** Corrosion rate of mild steel in 1 M HCl in the presence and absence of compounds A and B at different temperatures (exposure time 6 h)

Temp. (K)	Conc. (mM)	Corrosion rate (CR, mg cm^−2^ h^−1^)		*η*_W_%		*θ*	
		Comp. A	Comp. B	Comp. A	Comp. B	Comp. A	Comp. B
293	0	2.011	2.011				
	0.001	1.535	1.073	23.7	46.3	0.237	0.463
	0.01	0.980	0.457	51.3	77.3	0.513	0.773
	0.1	0.679	0.236	66.2	88.3	0.662	0.883
	0.25	0.469	0.205	76.7	89.8	0.767	0.898
	0.5	0.385	0.167	80.8	91.7	0.808	0.917
	1	0.294	0.135	85.4	93.3	0.854	0.933
	2	0.197	—	90.2	—	0.902	—
303	0	3.207	3.207				
	0.001	2.428	1.656	24.3	48.6	0.243	0.486
	0.01	1.496	0.535	53.4	83.2	0.534	0.832
	0.1	1.036	0.264	67.7	91.8	0.677	0.918
	0.25	0.727	0.221	77.3	93.1	0.773	0.931
	0.5	0.537	0.187	83.3	94.2	0.833	0.942
	1	0.386	0.140	88.0	95.6	0.880	0.956
	2	0.225	—	93.0	—	0.930	—
313	0	5.437	5.437				
	0.001	4.035	2.715	25.8	50.0	0.258	0.5
	0.01	2.491	0.785	54.2	85.4	0.542	0.854
	0.1	1.714	0.341	68.5	93.7	0.685	0.937
	0.25	1.189	0.235	78.1	95.6	0.781	0.956
	0.5	0.819	0.193	84.9	96.4	0.849	0.964
	1	0.604	0.153	88.9	97.2	0.889	0.972
	2	0.324	—	94.1	—	0.941	—
323	0	9.012	9.012				
	0.001	7.202	4.519	20.1	49.9	0.201	0.499
	0.01	4.511	1.471	49.9	83.7	0.499	0.837
	0.1	3.170	0.736	64.8	91.8	0.648	0.918
	0.25	2.385	0.603	73.5	93.31	0.735	0.933
	0.5	1.846	0.513	79.5	94.31	0.795	0.943
	1	1.478	0.355	83.6	96.0	0.836	0.960
	2	1.071	—	88.1	—	0.881	—

### Adsorption isotherm

**Fig. 5:**
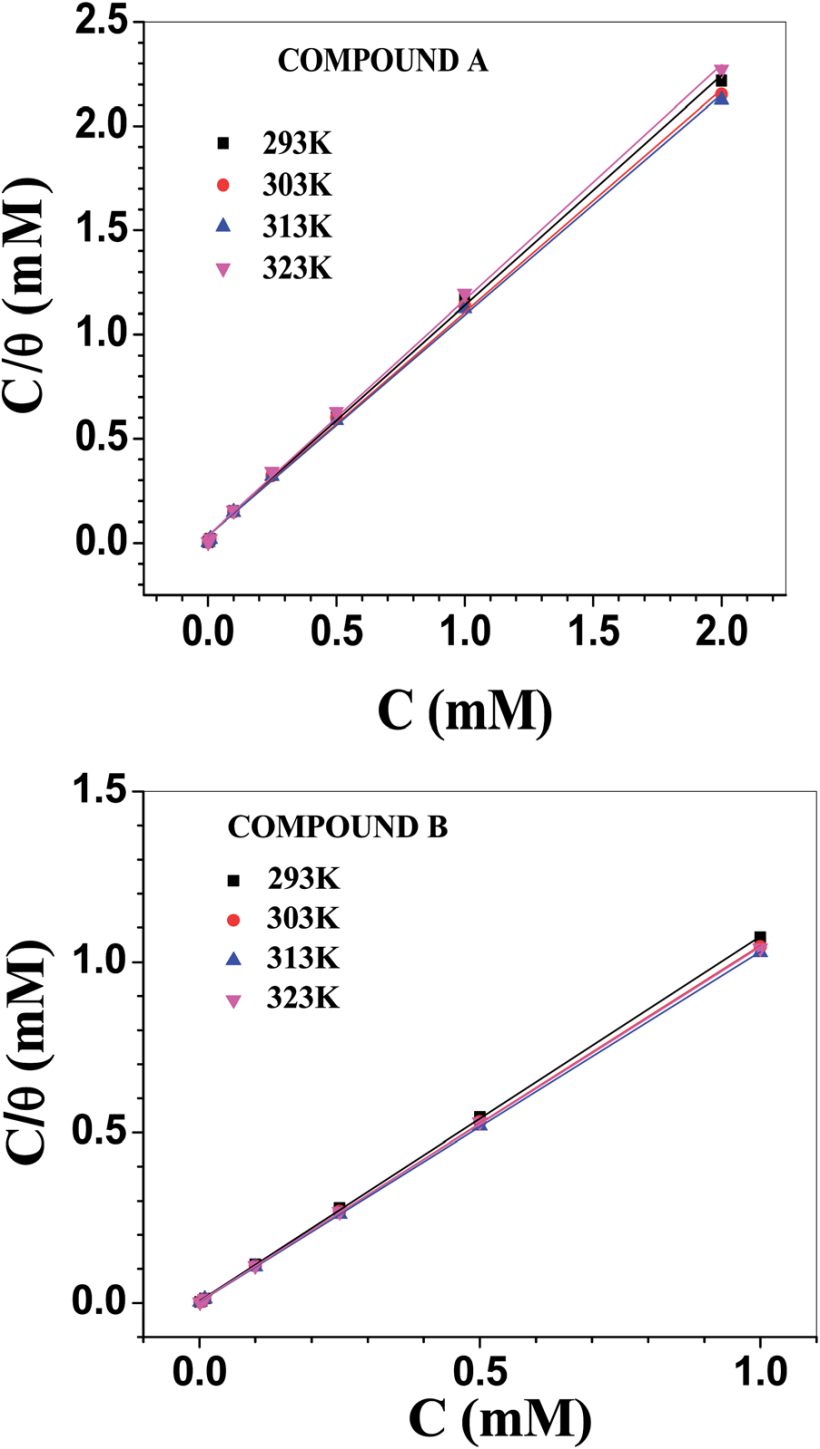
Langmuir adsorption isotherms involving compounds A (top) and B (bottom).

The Langmuir adsorption isotherm model was found to be best suited to describe the adsorption behaviour of the vanillin derivatives on the surface of mild steel in 1 M HCl (Fig. 5). The Langmuir adsorption isotherm, which relies on the monolayer formation hypothesis by energetically equivalent adsorption sites, is represented by the following equation:

20 *C*/*θ* = 1/*K*_ads_ + *C*

where *C* is the concentration of inhibitors used and *θ* the fraction of surface covered at the particular inhibitor concentration (as tabulated in Table 3). The free energy change in the adsorption (Δ*G*^0^_ads_) is related to the adsorption equilibrium constant (*K*_ads_) according to the following equation:

21 Δ*G*^0^_ads_ = −*RT* ln(55.56*K*_ads_)

where *R* is the universal gas constant, *T* is the experimental temperature and the factor 55.56 is the molarity of water (introduced as adsorption occurs in aqueous solution).^56^ For a broad range of degree of surface coverage, the nearly unity slope with a correlation coefficient (*r*^2^) value in the order 0.999 for all the plots of *C*/*θ* against *C* justifies the suitability of the Langmuir model in the present case. The reciprocal of the intercept of the plot of *C*/*θ vs. C* gives the *K*_ads_ and Δ*G*^0^_ads_ values according to eqn (21). These values are tabulated in Table 4. The other adsorption parameters such as the enthalpy of adsorption (Δ*H*^0^_ads_) and free energy of adsorption (Δ*S*^0^_ads_) were evaluated according to eqn (22) and tabulated in Table 5.

22 Δ*G*^0^_ads_ = Δ*H*^0^_ads_ − *T*Δ*S*^0^_ads_

**Table 4:** Adsorption parameters in the presence of compounds A and B at different temperatures

Temp. (K)	Slope		*K*_ads_ × 10^−3^ (mol^−1^)		Δ*G*^0^_ads_ (kJ mol^−1^)	
	Comp. A	Comp. B	Comp. A	Comp. B	Comp. A	Comp. B
293	1.104	1.070	28.65	186.92	34.79	39.35
303	1.070	1.045	28.09	253.16	35.92	41.46
313	1.058	1.028	28.98	355.87	37.19	43.72
323	1.129	1.049	27.62	235.85	38.25	44.01

**Table 5:** Δ*H*^0^_ads_ and Δ*S*^0^_ads_ data for compounds A and B

Temp. (K)	Δ*H*^0^_ads_ (kJ mol^−1^)		Δ*S*^0^_ads_ (J K^−1^ mol^−1^)	
	Comp. A	Comp. B	Comp. A	Comp. B
293–313	−0.656	24.7	116	218
313–323		−34.6		29

To construct the adsorption isotherms, the initial concentration of inhibitor was used rather than its equilibrium value. Hence, more importance should be given towards the relative variation in the adsorption constants and other adsorption parameters between the two inhibitors rather than their absolute values. The equilibrium adsorption constant was maximum at 313 K for both inhibitors and decreased thereafter. This indicates that the extent of adsorption is greater when induced by some degree of thermal energy. Further, the *K*_ads_ values for compound B are higher than that for compound A by many times and they are pronounced mostly at 313 K. For compound A, Δ*G*^0^_ads_ became gradually more negative with temperature, and the variation is linear in nature (Fig. S15 in ESI†). The corresponding heat and entropy of adsorption data is shown in Table 5, which reveals the exothermic nature of adsorption with a higher degree of randomness during adsorption. In contrast, for compound B, the dependency of Δ*G*^0^_ads_ on temperature is not linear over the whole temperature range. A break in the slope was observed between the lower and higher temperature ranges (Fig. S15 in ESI†). Accordingly, two sets of data for Δ*H*^0^_ads_ and Δ*S*^0^_ads_ were obtained in the lower and higher temperature ranges (Table 5). Inspection of Tables 4 and 5 reveals some important facts regarding the adsorption of the vanillin derivatives on the surface of mild steel in 1 M HCl. The Δ*G*^0^_ads_ values for compound B are more negative than that of compound A, suggesting the stronger adsorption of compound B. Further, the Δ*G*^0^_ads_ values for compound B decreased gradually with an increase in temperature, but rate of decrement was lower at a higher range of temperature (Fig. S15 in ESI†). This observed trend is characteristic of chemisorption, which is activated adsorption, and thus requires a minimum amount of energy to proceed. The variation in the adsorption constant with temperature also supports this view. Thus, up to a certain temperature range, adsorption remains essentially kinetically controlled. At sufficiently high temperature, adsorption becomes thermodynamically controlled and decreases with an increase in temperature.^21,57–60^ Δ*S*^0^_ads_ was positive for both inhibitors over the whole temperature range. This seems contrary to any adsorption process, which should result in a decrease in randomness. Thus, the results suggest that the removal of pre-adsorbed water molecules and other ions present in the electrolytic solution from the metal surface occurs simultaneously with the adsorption of the inhibitor molecules.^58^ The desorption of solvent molecules needs to be considered while explaining the variation in Δ*H*^0^_ads_. At a lower temperature range, it was positive for compound B, suggesting endothermic adsorption, whereas it became negative at a higher temperature. This observation can be interpreted similarly as that for Δ*S*^0^_ads_.

The literature suggests that the desorption energy (which is positive) of water molecules from the iron oxide surface varies from 50 to 100 kJ mol^−1^, depending on the temperature and limit of surface coverage.^61^ For compound A, the energy released for adsorption exceeds the energy required for the desorption of pre-adsorbed water molecules for the whole temperature range, making it an exothermic process. However, for the other inhibitor, at a lower temperature range, the opposite effect is more dominant, making the whole process endothermic. At an elevated temperature, the effect of adsorption becomes more dominant and exothermic adsorption behaviour is observed. According to the literature, chemisorption is accompanied with a heat of adsorption in the order of −100 kJ mol^−1^.^62^ Since the heat of desorption of pre-adsorbed water molecules also contributed during the evaluation of the heat of adsorption of the inhibitor molecules, the overall Δ*H*^0^_ads_ value calculated was much lower than that for the chemisorption process. However, the trend in the variation of the different adsorption parameters, as discussed above, conforms with the conclusion that the adsorption of the vanillin derivatives on the surface of mild steel in 1 M HCl is essentially chemisorption, with compound B interacting with the metal surface more strongly than compound A. Overall, the adsorption of compound A is both enthalpically and entropically favourable. For compound B, adsorption at a lower temperature is entropy driven, whereas at elevated temperature it is mostly enthalpy driven.

The change in the nature of adsorption for compound B going from lower to higher temperature (endothermic to exothermic behaviour) was observed for other systems also.^21,59^ Nevertheless, it still requires further experimentation at even higher temperatures to establish the phenomena unequivocally.

### Activation parameters

**Fig. 6:**
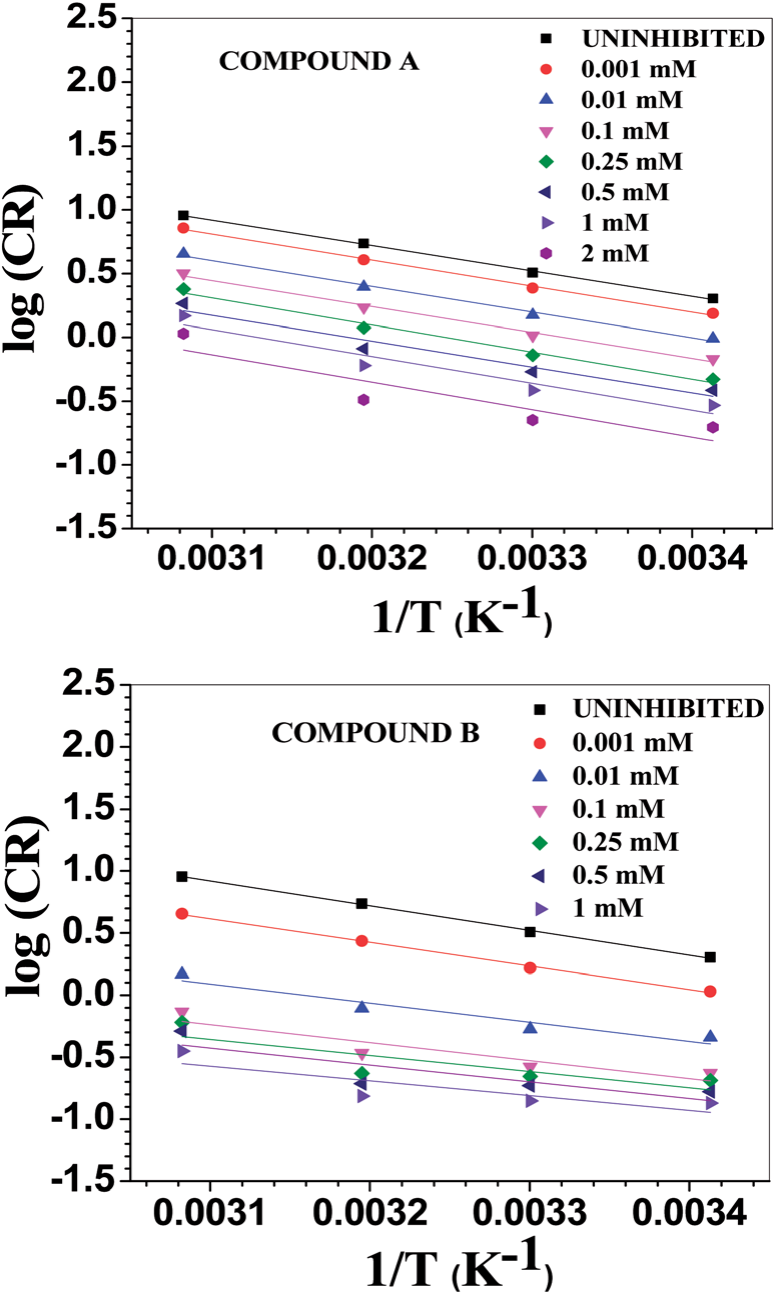
Arrhenius plots for mild steel in 1 M HCl with compounds A (top) and B (bottom).

To determine the activation parameters related to the corrosion process, we plotted the corrosion rate data against temperature using the following equations:^21,59^23
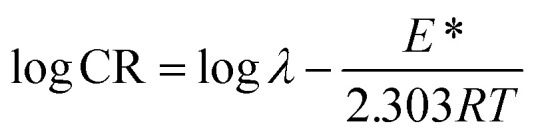
24
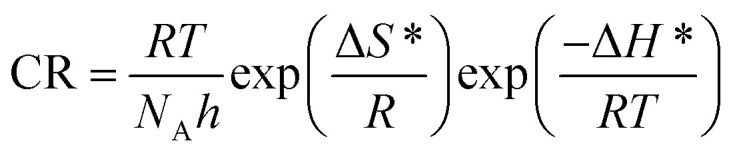
where *λ* is the pre-exponential factor (Arrhenius frequency factor), E* is the activation energy related to the corrosion process, *R* is the universal gas constant, *h* is Plank's constant, *N*_A_ is Avogadro's number, and *T* is the temperature. ΔS* and ΔH* are the entropy of the activation and enthalpy of activation, respectively. The plot of log(CR) *vs.* 1/*T* (Fig. 6) provides the value of *λ* and E* from the intercept and slope, respectively. On the other hand, ΔS* and ΔH* are calculated from the plot of log(CR/*T*) *vs.* 1/*T* (Fig. S16 in ESI†). From the slope of the plot, ΔH* can be calculated, and the intercept gives the value of ΔS*. The experimentally obtained data is presented in the Table 6.

**Table 6:** Activation parameters in the presence of compounds A and B

Conc. (mM)	*λ* × 10^−4^ (mg cm^−2^ h^−1^)		E* (kJ mol^−1^)		ΔH* (kJ mol^−1^)		ΔS* (J K^−1^ mol^−1^)	
	Comp. A	Comp. B	Comp. A	Comp. B	Comp. A	Comp. B	Comp. A	Comp. B
Uninhibited	1212.83	1212.83	38.09	38.09	36.97	36.97	−113.1	−113.1
0.001	1318.56	323.07	38.98	36.40	37.85	35.21	−112.4	−124.4
0.01	697.11	7.23	38.55	29.46	37.38	27.79	−117.9	−157.5
0.10	542.13	1.86	38.84	27.83	37.67	25.97	−119.9	−169.4
0.25	788.32	0.49	40.68	25.00	39.54	22.87	−116.8	−181.4
0.50	284.90	0.59	38.80	25.93	37.45	23.82	−125.9	−179.8
1.00	366.61	0.13	40.17	22.76	38.81	20.60	−123.8	−192.6
2.0	348.34	—	41.24	—	39.66	—	−125.0	—

The activation parameters calculated from the corrosion rate data using the weight loss method give valuable information regarding the probable mechanism of adsorption of the inhibitor on the metal surface. It was observed that the activation energy in the presence of compound A was slightly higher than that of the uninhibited sample, while the presence of compound B resulted in a decrease in the activation energy compared to that of the uninhibited specimen to a significant extent. The Arrhenius equation suggests that the variation in the reaction rate with temperature will be more with a higher activation energy. This explains the observation regarding the relative variation in the reaction rate with temperature in the absence and presence of the inhibitors. For the uninhibited sample and in the presence of compound A, the rate of corrosion increased to a greater extent with an increase in temperature than that with compound B. Furthermore, it is argued that when the values of E* and ΔH* of the inhibited system is comparable or lower than that of the uninhibited system, the adsorption may be predicted to be chemisorption, whereas a higher value of E* and ΔH* of the inhibited system compared to that of the uninhibited system leads to physisorption.^21,59,63^ Inspection of the table leads to the conclusion that chemisorption is preferred over physisorption. The rate of the decrease in corrosion with an increase in inhibitor concentration can be explained by the decreased value of the pre-exponential frequency factor. Again, according to Table 6, ΔS* is negative for the uninhibited system, and it is even more negative in the presence of the inhibitors. This means that the transition state in the corrosion process, which is the rate-determining step, is more ordered than the reactants, and the degree of order of the transition state is higher when an inhibitor layer is present on the metal surface. This can be explained considering the detailed mechanism of the hydrogen evolution reaction (eqn (25)–(27)) together with the adsorption of the inhibitor on the metal surface.^64,65^

25 M + H_3_O^+^ + e^−^ → MH_ads_ + H_2_O

26 2MH_ads_ ↔ 2M + H_2_

27 MH_ads_ + H_3_O^+^ + e^−^ ↔ M + H_2_ + H_2_O

Any one of these intermediate steps may be the rate-determining step, depending on the nature of the catalytic surface and other reaction conditions. In general, when the cathodic Tafel slope is close to −120 mV dec^−1^ for Fe in acidic solution, the first reaction, which is a surface-catalyzed charged transfer reaction and designated as the Volmer reaction, is considered to be the rate determining step.^65^ Following the cathodic Tafel slope tabulated in Table 1, we may presume that in the present case, the reaction shown in eqn (25) is the rate-determining step. In fact, all these intermediate steps lead to a decrease in the randomness in their corresponding transition states, which is reflected in the negative value for ΔS*. Again, the more negative value of ΔS* in the presence of the inhibitor compared to that of the uninhibited specimen can be explained in terms of the blocking of the cathodic and anodic reaction sites by the inhibitor molecules. For occurrence of any type of surface reaction, reactants require closer approach or interaction with a metal surface possessing an inhibitor layer, resulting in more order during the intermediate stages of the reaction. This also results in a decrease in the pre-exponential frequency factor.^1^

The Arrhenius plots show a deviation from linearity in the case of higher inhibitor concentrations. This non-Arrhenius behavior is observed for complex processes when the mechanism of the reaction, and hence the activation parameters, depends on temperature.^66^ In case of the inhibitor-adsorbed metal surface, the extent of adsorption depends on the temperature. This will influence the mechanism of the corrosion process to a certain extent and will be the guiding factor for the non-linearity observed in Fig. 6.

### Comparison of the inhibitory action of the inhibitors

Since compound B is essentially the dimeric form of compound A, one should expect a similar corrosion inhibitory effect of compound B at half of the concentration of compound A. However, the analysis of the corrosion current density (*i*_corr_) values reveals that the *i*_corr_ at 0.5 mM concentration of compound A is 675 μA cm^−2^, while that at 0.25 mM concentration of compound B is 280 μA cm^−2^ (Table 1). This is much lower than the half of the other value. Similarly, the *R*_p_ value in the presence of 0.5 mM compound A is 22.6 ohm cm^2^, whereas for 0.25 mM compound B, it is 63 ohm cm^2^ (Table 2). Thus, the inhibitory effect for compound B is more than twice that of compound A, and this trend is valid throughout the lower concentration range. A similar conclusion can be derived from the weight loss measurements (Table 3). When we compared the *i*_corr_ and *R*_p_ values for 2 mM compound A and 1 mM compound B, the inhibitory effect was still higher for compound B, but it not twice with respect to the other. This suggests a leveling effect at a higher concentration towards the corrosion inhibitory property, which originates from the fact that at higher concentration, the metal surface is almost fully covered by the inhibitors, and therefore they exert very comparable inhibitory action. The better corrosion inhibitory propensity of compound B over compound A can be explained from the result obtained from the DFT calculation.

### SEM morphology

**Fig. 7:**
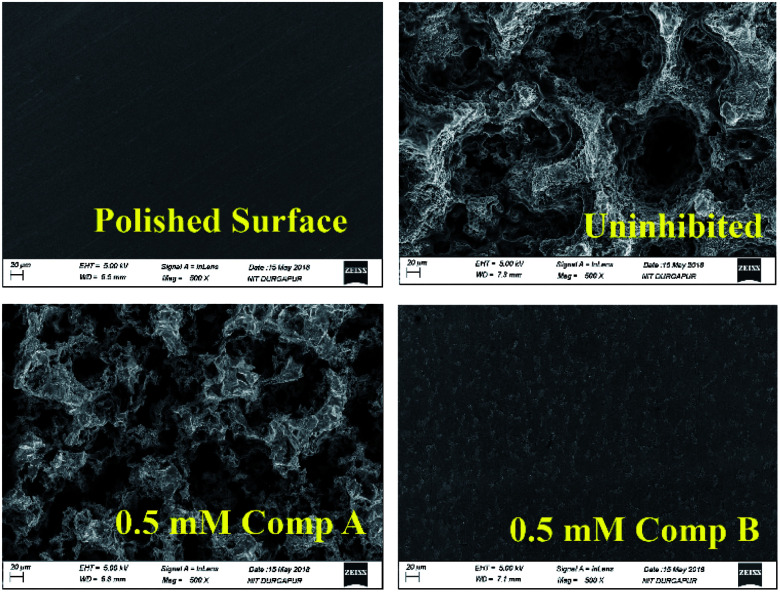
SEM images of the surface of the mild steel sample after immersing it for 6 h in 1 M HCl at room temperature.

By comparing the SEM images of the uninhibited and inhibited (0.5 mM of compounds A and B) steel samples after immersion in 1 M HCl medium for 6 h, the anti-corrosive activity of the studied Schiff base derivatives was determined. In the presence of compound B, the metal surface was corroded less, reflecting the better corrosion mitigation propensity of compound B over that of compound A for mild steel in aqueous HCl (Fig. 7).

### DFT study results

**Fig. 8:**
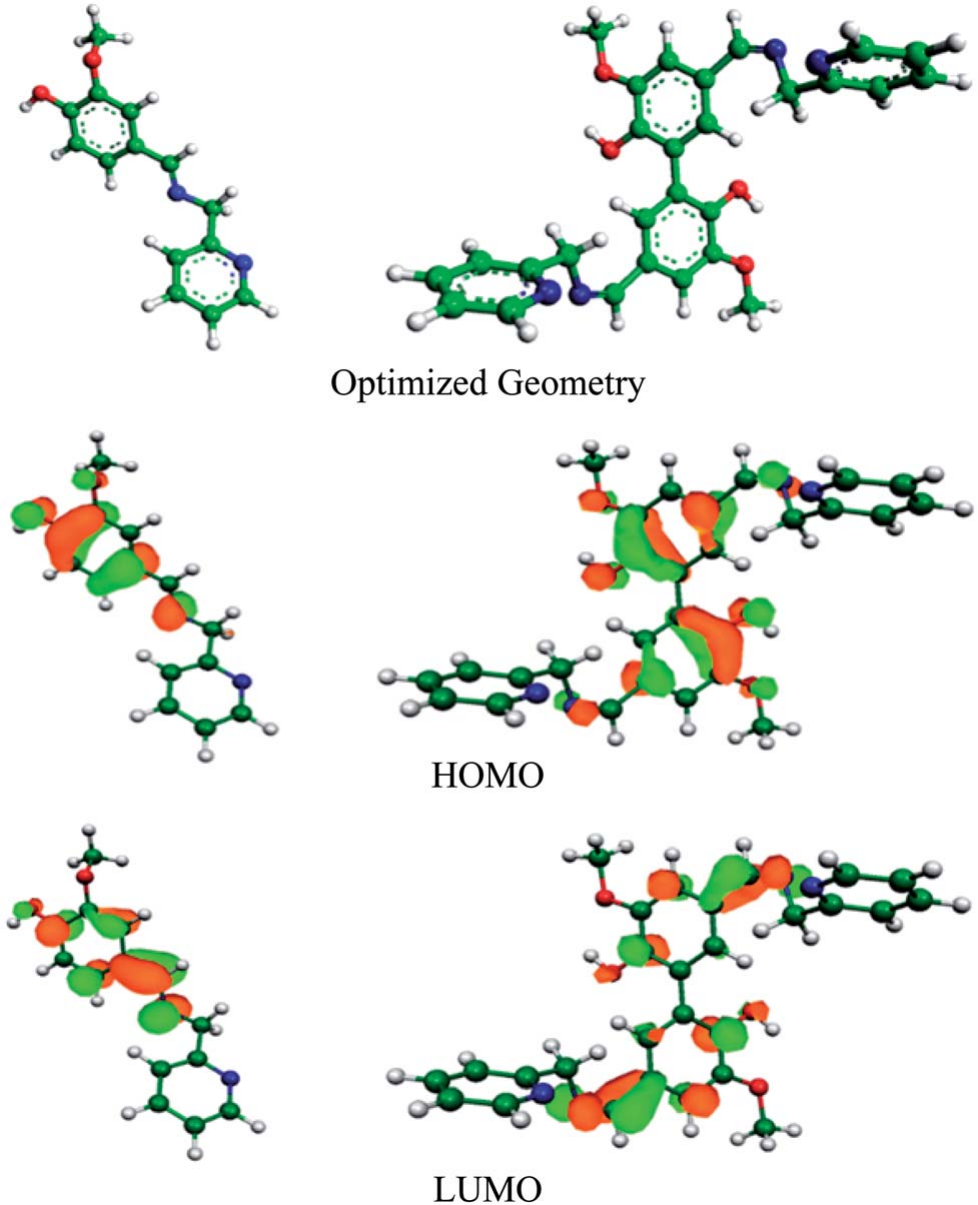
Optimized geometry and electron distribution in the HOMO and LUMO of compounds A (left) and B (right) as obtained from the DFT study.

DFT study can be used to explain the nature of bonding between the organic inhibitor molecules and metal. Electron transfer from the inhibitor to the vacant 3d orbitals of Fe occurs through the HOMO of inhibitor, whereas its LUMO is responsible for retro-electron transfer from the filled 4s metal orbital.^15,67^ The higher the energy of the HOMO and the lower the energy of the LUMO favour the forward and back electron donation, respectively. Table 7 provides details of the quantum chemical parameters of the inhibitors studied. Fig. 8, S17 and S18 (in the ESI†) show the energy-optimized spatial configurations of the inhibitors and the electronic distribution in the HOMO and LUMOs of the inhibitors. It was observed that when vanillin is conjugated with picolylamine through imine bond formation (*i.e.* compound A), both *E*_HOMO_ and *E*_LUMO_ increase from that of vanillin and picolylamine. Thus, the observed higher inhibition efficiency of compound A compared to vanillin and picolylamine may be attributed to the more facile forward electron transfer for compound A than the retro-electron transfer. This is reflected by the fraction of electrons transferred from the inhibitor to the metal (Δ*N*). According to the electronic distribution in the HOMO of vanillin, the electron cloud is distributed over the whole molecular surface, including the phenyl ring and the oxygen atoms present in the substituent groups attached to it. In compound A, it is extended up to the imine group present. This elaborates the specific role of the imine bond present in compound A towards its corrosion inhibition propensity. Thus, it can be concluded that the better forward electron donation from compound A to the metal and the involvement of the imine group are the main reasons for the greater inhibition efficacy of compound A compared to its precursor molecules.

**Table 7:** Calculated molecular parameters from the DFT study

Inhibitors	*E*_HOMO_ (eV)	*E*_LUMO_ (eV)	Δ*E* (eV)	*μ* (D)	*I* (eV)	*A* (eV)	*X* (eV)	*η* (eV)	*σ* (eV^−1^)	Δ*N*
**Neutral form**										
Compound A	−6.1852	−1.3545	4.8307	3.37	6.1852	1.3545	3.7698	2.4154	0.414	0.217
Compound B	−6.0556	−1.4355	4.6201	4.43	6.0556	1.4355	3.7456	2.3101	0.433	0.232
Vanillin	−6.5700	−1.8976	4.6724	5.55	6.5700	1.8976	4.2338	2.3362	0.428	0.125
Picolyl amine	−6.8094	−0.9280	5.8814	4.50	6.8094	0.9280	3.8687	2.9407	0.340	0.161

**Protonated form**										
Compound A	−6.8999	−1.9273	4.9726	23.37	6.8999	1.9273	4.4136	2.4863	0.402	0.082
Compound B	−7.2086	−2.4362	4.7724	29.91	7.2086	2.4362	4.8224	2.3862	0.419	−0.0005

Comparing compounds A and B, it is evident that the HOMO energy is higher for compound B, whereas the LUMO energy is lower for the dimeric form (compound B) than the monomeric form (compound A). Thus, two-way electron transfer (inhibitor to the metal and *vice versa*) is more facilitated for compound B than compound A. This resulted in a higher softness, *σ*, and higher fraction of electrons transferred, Δ*N*, for compound B compared to compound A. The higher softness of compound B makes it more susceptible towards charge transfer. Furthermore, the dipole moment of compound B is greater than that of compound A. Thus, the dimeric form is more prone to electrostatic interaction. All these favourable molecular parameters make compound B the better corrosion inhibitor than compound A. Analysis of the electronic distribution in the HOMO and LUMO for both compounds revealed some interesting information regarding the possible mode of interaction between the inhibitors and the metal. In compound B, two vanillin moieties lie in a planar orientation. The HOMO and LUMO in compound A are distributed mostly over the vanillin moiety and the imine group. In contrast, for compound B, these encompass both the vanillin moieties and the two imine groups attached with vanillin moieties. This implies that if compound A is capable of interacting with either the cathodic or anodic reaction site one at a time, compound B can interact with both reaction sites simultaneously. This will lead to the cooperative mode of interaction for the two vanillin moieties present in compound B. When one moiety donates electrons from its HOMO to the anodic reaction site (thereby reducing the rate of the metal oxidation reaction by enhancing the electron density in the anodic site), its ability to take up electrons in its LUMO from the cathodic reaction site of the metal increases, thereby reducing the rate of the hydrogen evolution reaction at the cathodic site to a greater extent. The reverse phenomenon also has equal possibility. Thus, both compounds essentially act as mixed-type inhibitors, but compound B, by virtue of the cooperative interaction between its two vanillin moieties, exhibits better proficiency towards the mitigation of corrosion.^19,39,68^

### Local reactivity analysis

To establish the local reactivity of the atoms present in compounds A and B against nucleophile and electrophile attack, positive and negative Fukui indices (*i.e.*, *f*^+^_k_ and *f*^−^_k_) values for the individual atoms were calculated and the data is presented in Table S6 (in ESI†). This data clearly shows that the O atoms attached to the benzene rings (*i.e.*, O atoms of the –OH and OCH_3_ substituents attached to the benzene group), C atoms of the benzene rings and N atom of the imine group have higher *f*^+^_k_ and *f*^−^_k_ values for both compounds. Thus, the vanillin moiety present in these compounds together with the imine bond are mostly involved in electrophilic and nucleophilic reactions. This tallies totally with the conclusion derived from the electronic distribution in the HOMO and LUMO levels obtained from the DFT calculation.

### DFT study result with protonated form of inhibitors

The local reactivity analysis revealed that the O atom of the –OH group attached to the benzene ring (O-3 in compound A and O-32 and 34 in compound B, Table S6 in the ESI†) possess the maximum *f*^−^_k_ value, making them the most prone towards protonation, *i.e.* attack from an electrophile, H^+^. In highly acidic medium, it is expected that any organic molecule having heteroatoms with free lone pairs of electrons should remain in the protonated form. Accordingly, we performed the DFT calculation with monoprotonated compound A and diprotonated compound B. It was observed that protonation does not alter the electronic distribution to any appreciable extent in the HOMO and LUMO levels for compound A (Fig. S18 in the ESI†), which are mostly distributed over the vanillin moiety and imine group. Thus, the interaction pattern for the neutral and protonated compound A should more or less be the same with the metal surface in acidic medium. For compound B, there is definite change in the possible interaction mode (Fig. S18 in the ESI†). The electronic distribution in the HOMO is localized mostly on the imine and pyridine groups. The LUMO is distributed all over the divanillin moiety, similar to that for the neutral state. Here, according to the molecular parameters, it was observed that protonation results in a decrease in both *E*_HOMO_ and *E*_LUMO_. This suggests that both compounds in their protonated state are more reactive for acceptance of electrons from the metal surface rather than forward electron transfer. As a result, value of the fraction of electrons transferred, Δ*N*, decreases and even becomes negative for compound B. This is contrary to the fact that experimentally, compound B provides much better corrosion inhibition potentiality than compound A. This supports the conclusion that despite the existence of the protonated form of the inhibitor molecules in acidic aqueous medium, during interaction with the metal surface, the neutral form of the molecules is mostly involved. This conclusion is also supported from the experimental observation that the formation constants of the Schiff base–metal ion complexes are greater than that of the corresponding protonated Schiff bases.^69^ A similar conclusion was derived for other heterocyclic bases.^9,10,34^

### Possible mode of interaction between the inhibitor molecules and metal surface

**Fig. 9:**
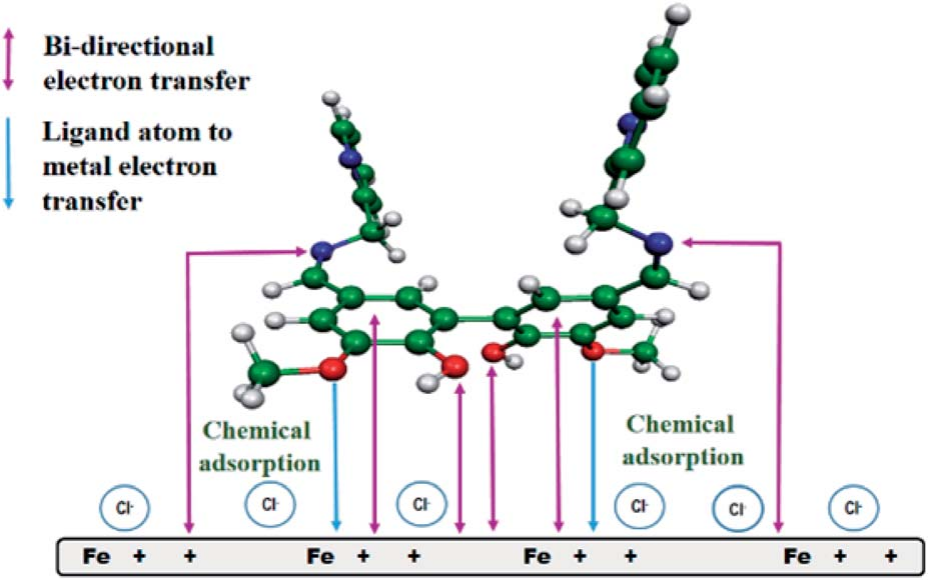
Pictorial representation of the adsorption of compound B on the surface of mild steel in 1 M HCl medium.

From all the above observations, we proposed a plausible model for the mode of adsorption of the studied Schiff bases on the surface of mild steel in acidic medium. It was experimentally verified that the surface of mild steel bears an excess positive charge in 1 M HCl at the equilibrium potential.^11^ Chloride ions and water molecules form an adsorbed layer on the positively charged metal surface.^11^ The protonated Schiff bases are attracted to the metal surface by the surface adsorbed chloride ions. When these inhibitor molecules come sufficiently close to the positively charged metal surface to form a chemisorbed layer, the Schiff bases are deprotonated and interact directly with the metal surface, replacing the pre-adsorbed chloride and water molecules. The inhibitor molecules through the vanillin moiety and the imine group donate electrons to the anodic sites on the metal, whereas these same groups attract electrons from the cathodic sites (Fig. 9). For compound A, electron donation and acceptance involve two different inhibitor molecules, whereas for compound B (the dimeric form of compound A), a single molecule is capable of bi-directional electron transfer. Electron donation and acceptance in a single molecule reinforce each other, and is the basis for the observed superior corrosion protection ability by compound B.

### MD simulation result

**Fig. 10:**
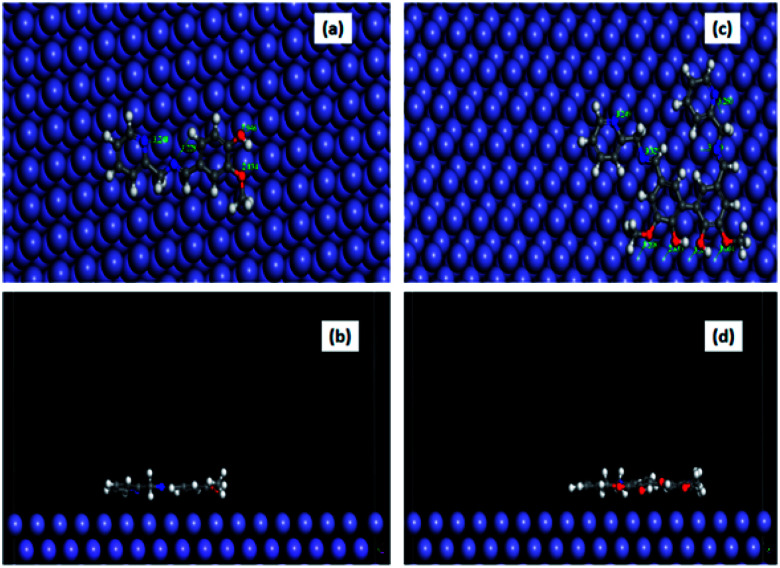
Equilibrium adsorption configurations of compound A (a and b) and compound B (c and d) on the Fe (1 1 0) surface obtained by molecular dynamics simulation. Top: top view and Bottom: side view (water molecules are not shown for clarity).

The equilibrium configuration of compounds A and B on the Fe (1 1 0) surface in aqueous HCl medium as obtained from the molecular dynamics simulation study is shown in Fig. 10, and the corresponding interaction energy data is tabulated in Table 8. The interaction energy for compound B was found to be almost twice that of compound A. This verifies the experimental observation that compound B has stronger susceptibility to interact with the Fe surface in 1 M aqueous HCl. Furthermore, we estimated the closeness of approach of various atoms present in both inhibitors with the metal surface in the equilibrated state (Table S7 in the ESI†). It was revealed that the O atoms present in the substituted –OH and –OCH_3_ groups and N atoms of the imine and pyridine groups form a distance with the Fe surface in the range of 2.8 to 3.8 Å. This is close enough to conclude that the inhibitor molecules form a chemisorbed layer on the metal surface.^1,15,35^ A similar conclusion was derived based on the calculated kinetic–thermodynamic parameters.

**Table 8:** Interaction energies of compound A and compound B on the Fe (1 1 0) surface

System	*E*_interaction_ (kJ mol^−1^)
Fe + compound A	−625.19
Fe + compound B	−1199.05

## Conclusion

Vanillin is an important edible flavour and available from bio-resources. Vanillin and its dimer, divanillin, were derivatized with picolyl amine into the corresponding Schiff base compounds.

The adsorption behaviour and anti-corrosion potential of these compounds towards mild steel in 1 M aqueous HCl were compared. The main conclusions of our work are summarized below.

• Compounds A and B (Schiff bases of vanillin and divanillin, respectively) bestow good protection to mild steel from corrosion in 1 M HCl medium from room temperature to as high as 323 K at the mM concentration level.

• These Schiff base derivatives act as mixed-type corrosion inhibitors, as evident from the potentiodynamic polarisation study.

• The extent of the adsorption and corrosion inhibition propensity of compound B is more than twice that of compound A at a lower concentration level.

• These molecules are adsorbed on the metal surface by chemical mode of adsorption through the vanillin moiety and imine group.

• The entire adsorption process is governed by both enthalpy and entropy changes.

• Due to its higher HOMO and lower LUMO energy, compound B acts as the better corrosion inhibitor. The other intrinsic molecular parameters such as global softness and dipole moment also impart a favourable impact during the interaction of compound B with the surface of mild steel in aqueous HCl.

• The Langmuir adsorption model can be suitably applied to calculate the different thermodynamic parameters related to the adsorption process.

• The interaction energy obtained from the molecular dynamics simulation is nearly two times higher for compound B than compound A, revealing that the simulation method is an important theoretical tool to compare the anti-corrosive behaviour of structurally comparable corrosion inhibitors.

## Conflicts of interest

There are no conflicts to declare.

## Supplementary Material

RA-010-C9RA07982C-s001
