# Supplementary material for: Adsorption and anti-corrosion characteristics of vanillin Schiff bases on mild steel in 1 M HCl: experimental and theoretical study
Source: RSC Adv. 2020 Mar 5;10(16):9258–73. doi: 10.1039/c9ra07982c (PMC9050057; doi:10.1039/c9ra07982c)
Supplement: RA-010-C9RA07982C-s001 [file RA-010-C9RA07982C-s001.pdf]

Electronic Supplementary Material (ESI) for RSC Advances.
This journal is © The Royal Society of Chemistry 2020

SUPPLEMENTARY INFORMATION

Adsorption and anti-corrosion characteristics of Vanillin Schiff bases on mild steel in 1M HCl: Experimental and theoretical study

Sanjoy Satpati,[#] Sourav K. Saha,[§] Aditya Suhasaria,[#] Priyabrata Banerjee,[§*] Dipankar Sukul[#*]

[#]Department of Chemistry, National Institute of Technology, Durgapur, West Bengal, 713209, India. E-mail: dipankar.sukul@ch.nitdgp.ac.in; Tel: +91 9434788066

[§] Surface Engineering & Tribology Group, CSIR-Central Mechanical Engineering Research Institute, Durgapur 713209, India.

[§]Academy of Scientific and Innovative Research, CSIR-CMERI Campus, Durgapur 713209, India. E-mail: pr_banerjee@cmeri.res.in (P. Banerjee); Tel: +91 343-6452220

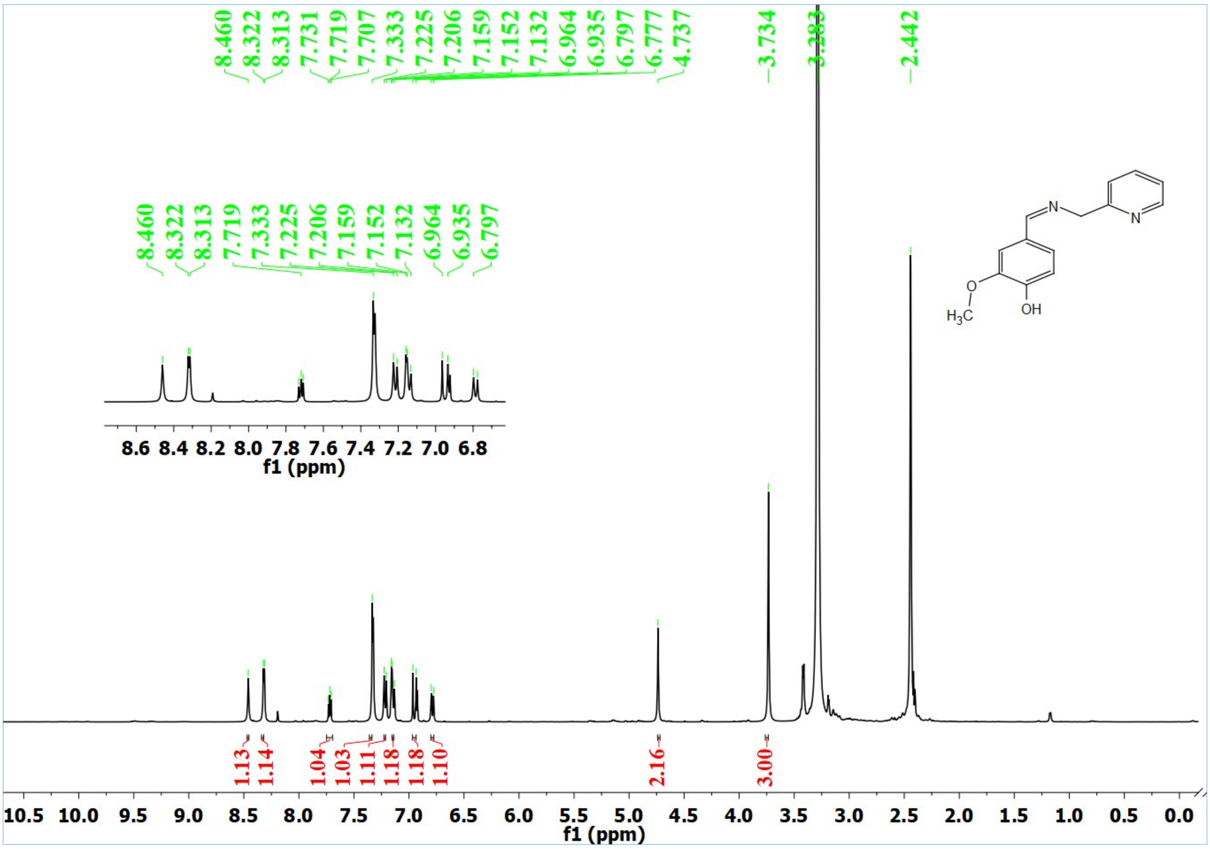

Fig. S1 ^{1}H NMR of Compound A

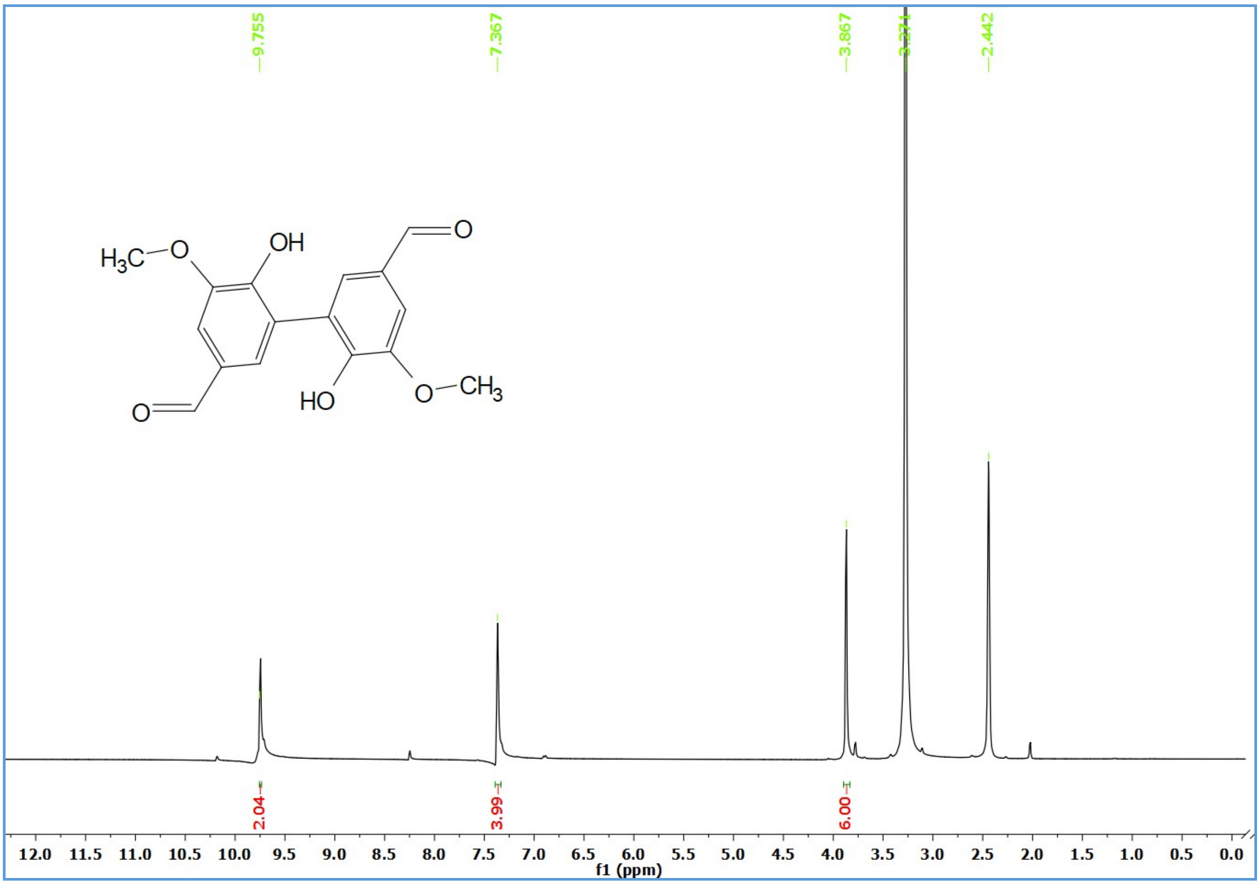

Fig. S2 ^{1}H NMR of divanillin

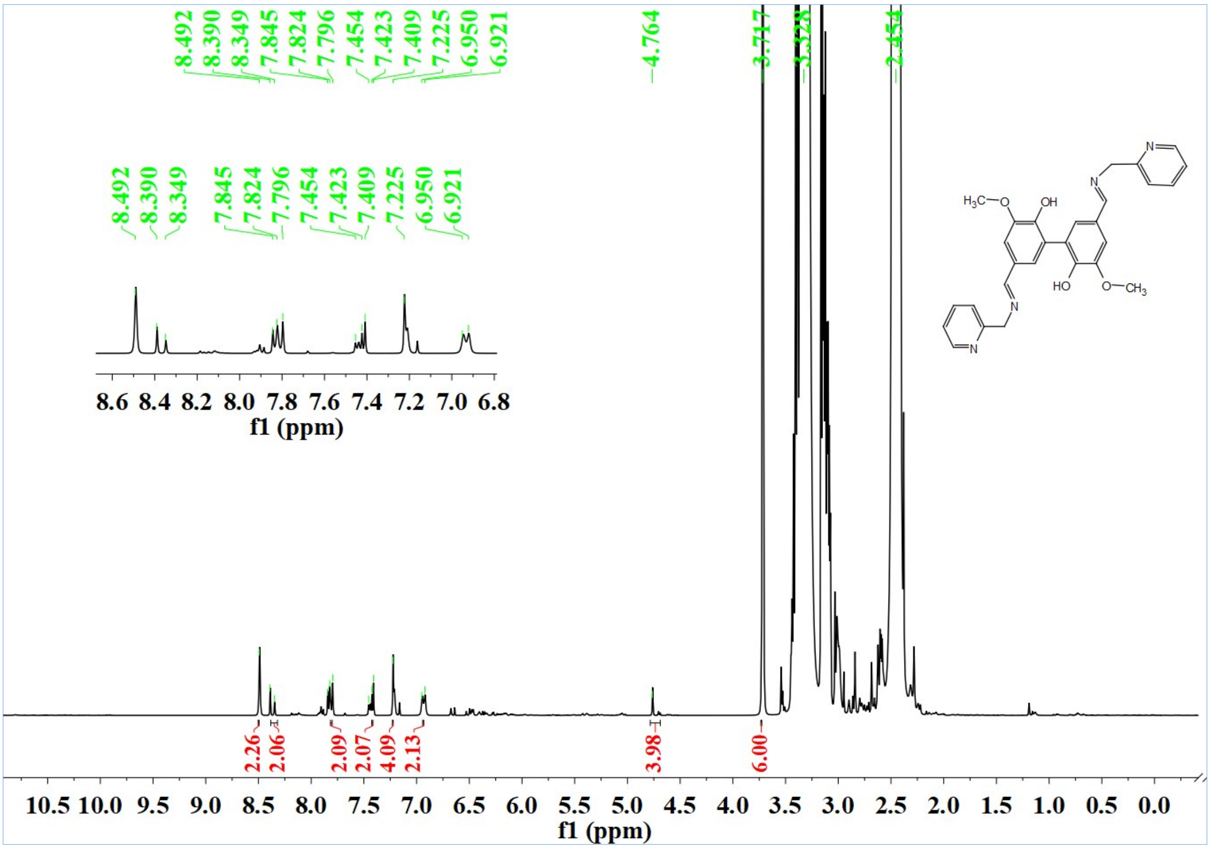

Fig. S3 ^{1}H NMR of Compound B

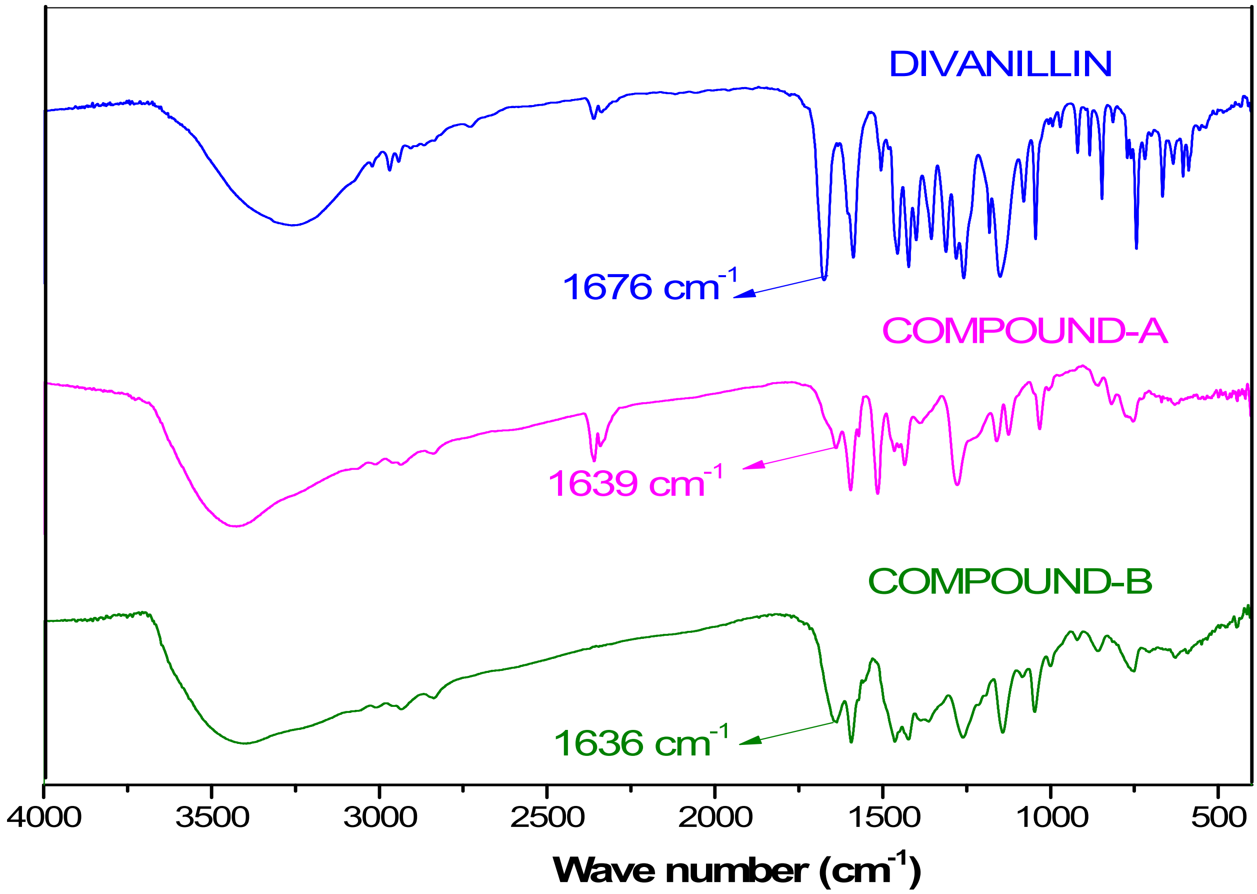

Fig. S4 FTIR spectra of divanillin, Compound A and Compound B

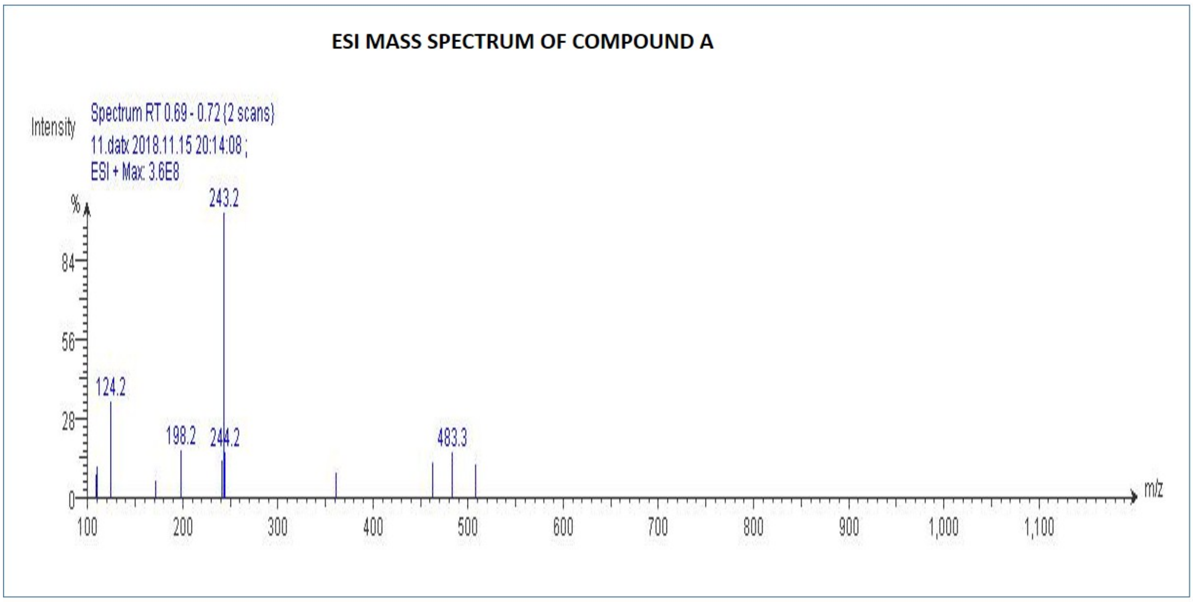

Fig. S5 ESI-Mass spectrum of Compound A

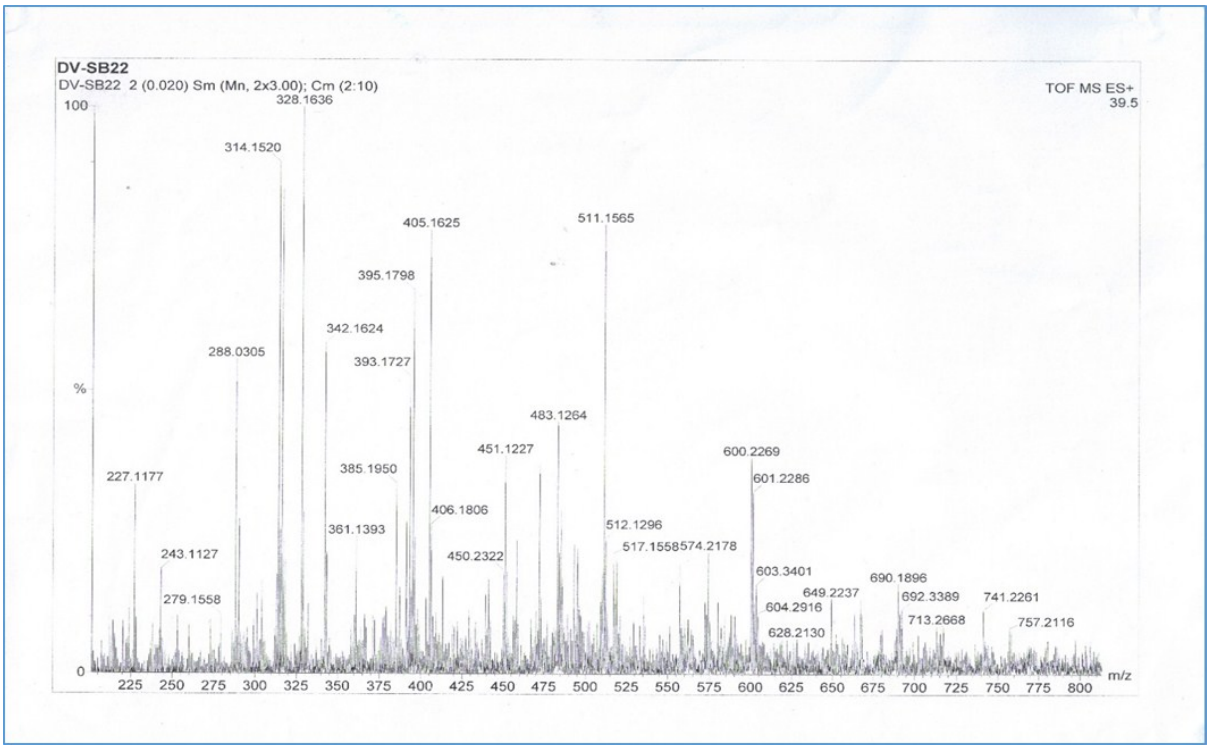

Fig. S6 ESI-Mass spectrum of Compound B

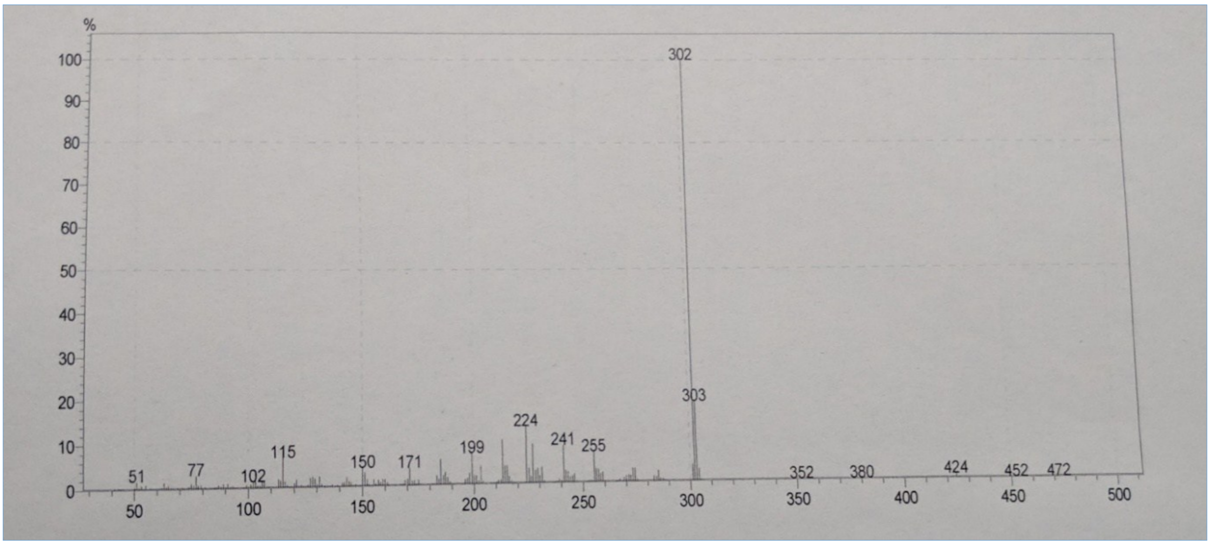

Fig. S7 EI-Mass spectrum of divanillin

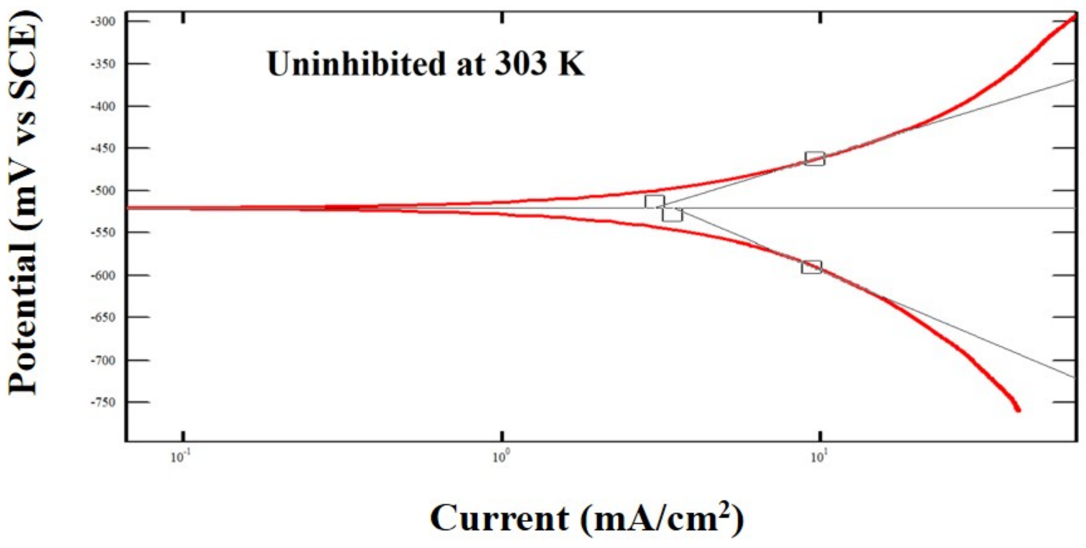

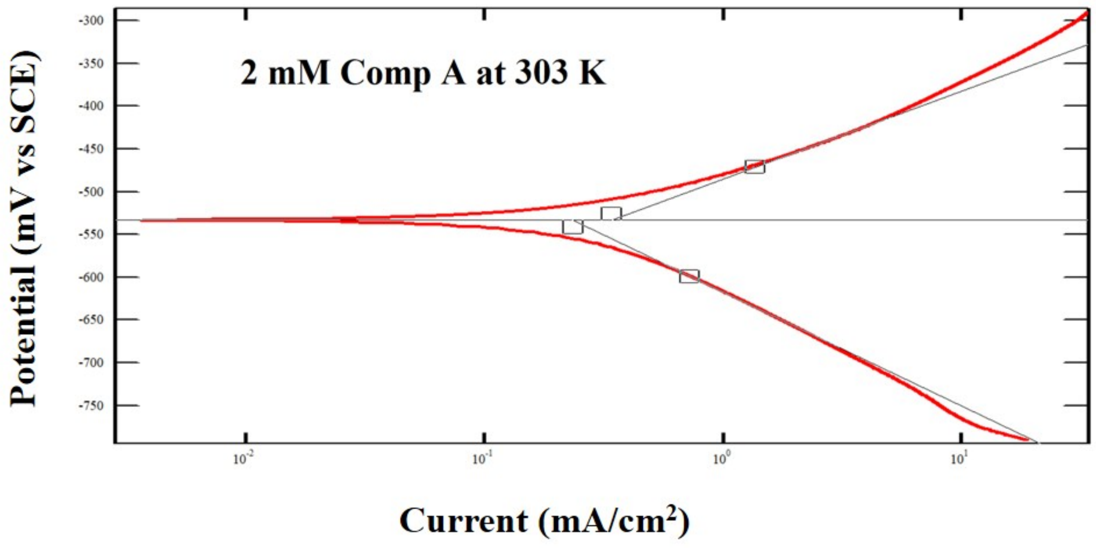

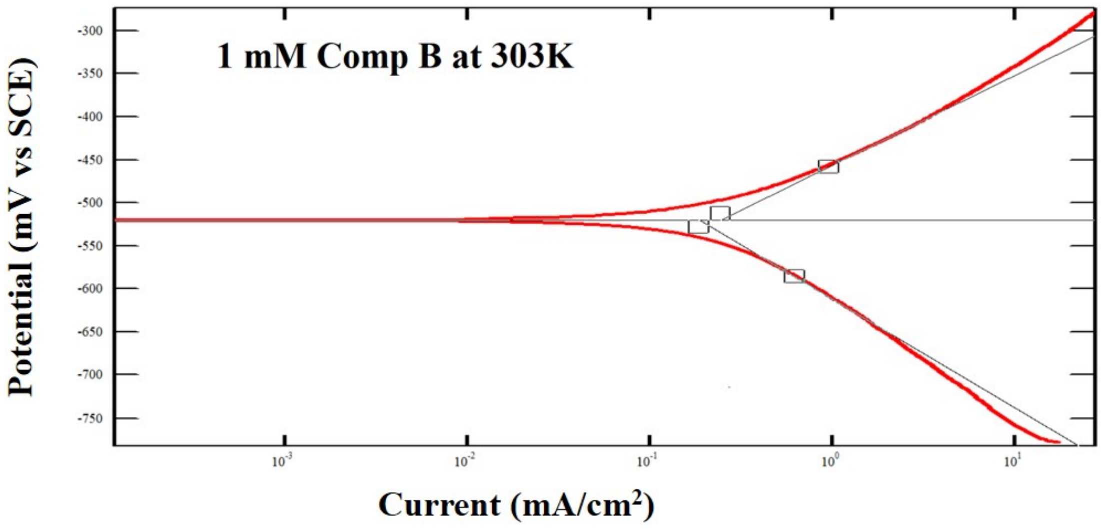

Fig. S8 Fitting of the Potentiodynamic polarization plots of uninhibited (top) and inhibited (middle and lower figures represent 1mM of Comp A and B respectively) samples at 303K.

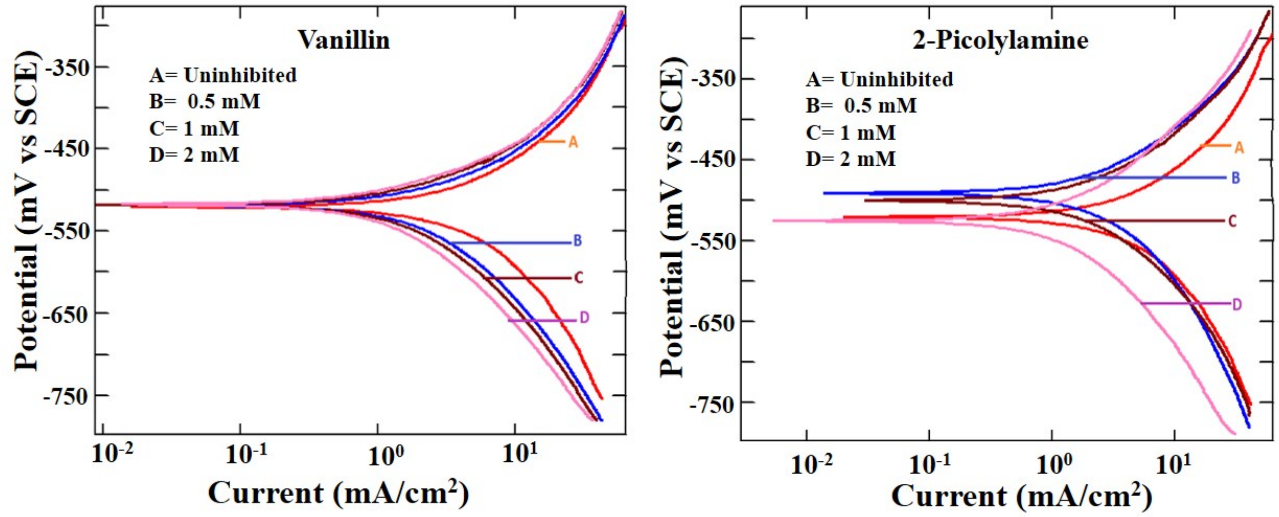

Fig. S9 Potentiodynamic polarization plots for mild steel in 1 M HCl in presence of vanillin (left) and 2-picolylamine (right) at 303K.

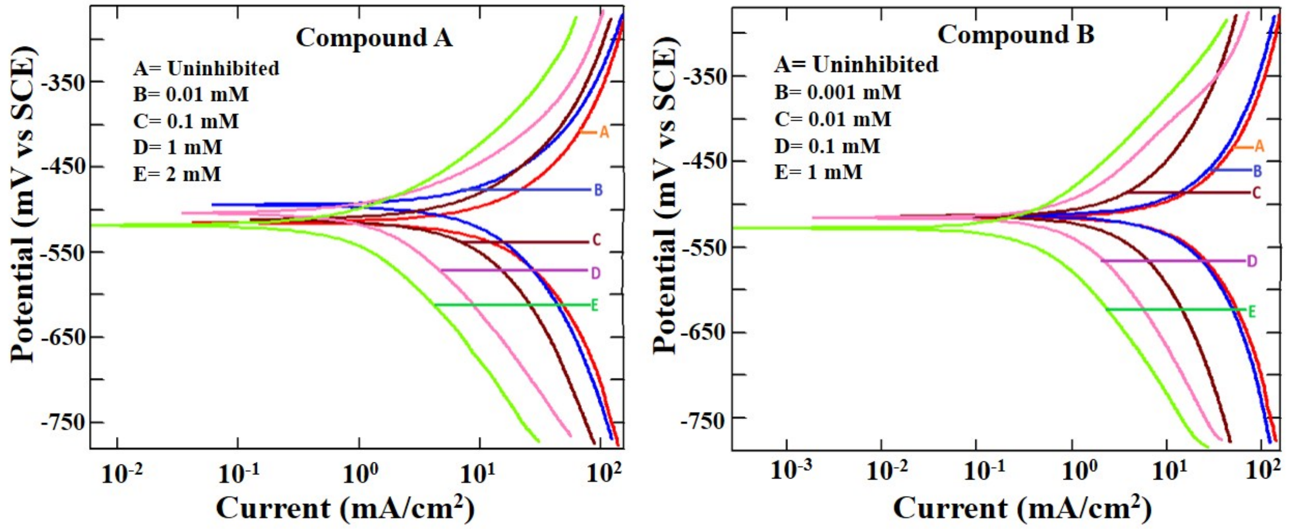

Fig. S10 Potentiodynamic polarization plots for mild steel in 1 M HCl in presence of compound A (left) and compound B (right) at 323K

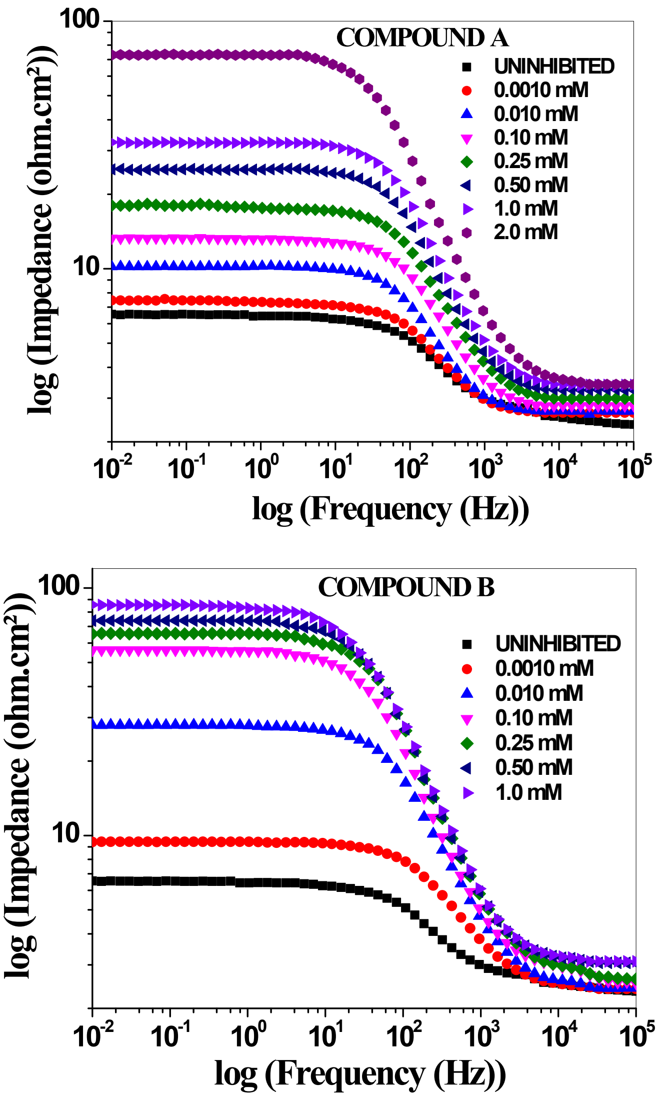

Fig. S11 Bode impedance plots in absence and presence of compound A and compound B at 303K

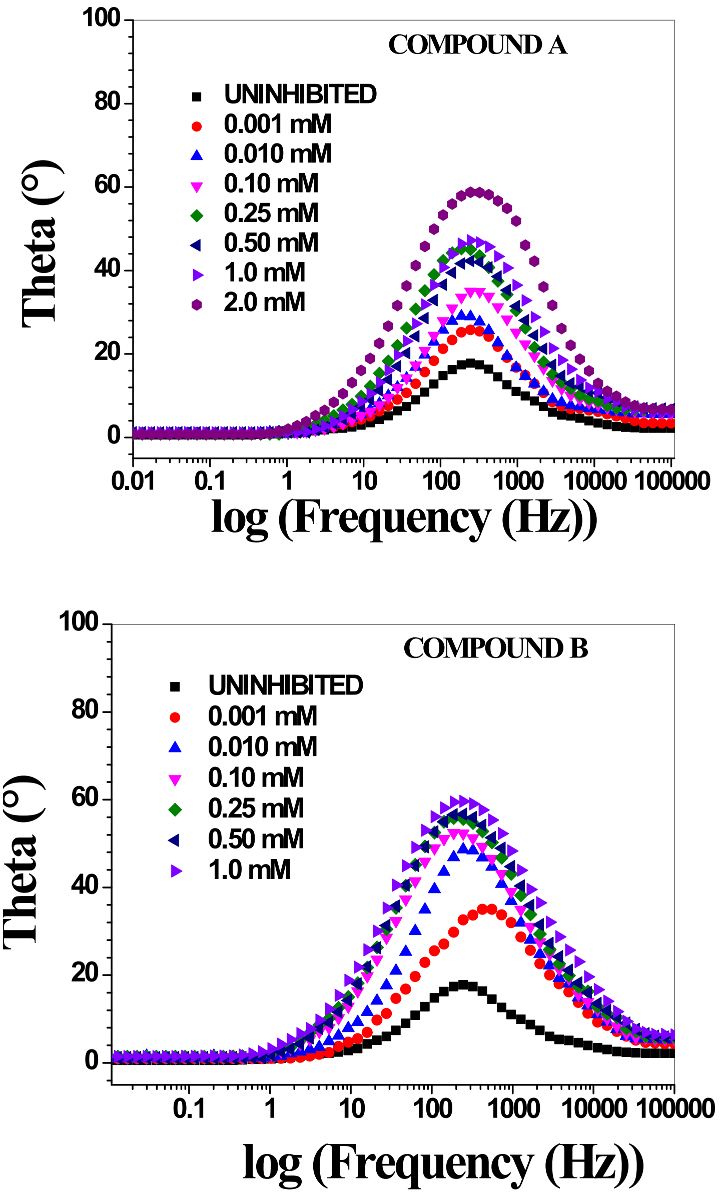

Fig. S12 Bode phase angle plots in absence and presence of compound A and compound B at 303K. Inset shows the corresponding plot for uninhibited sample.

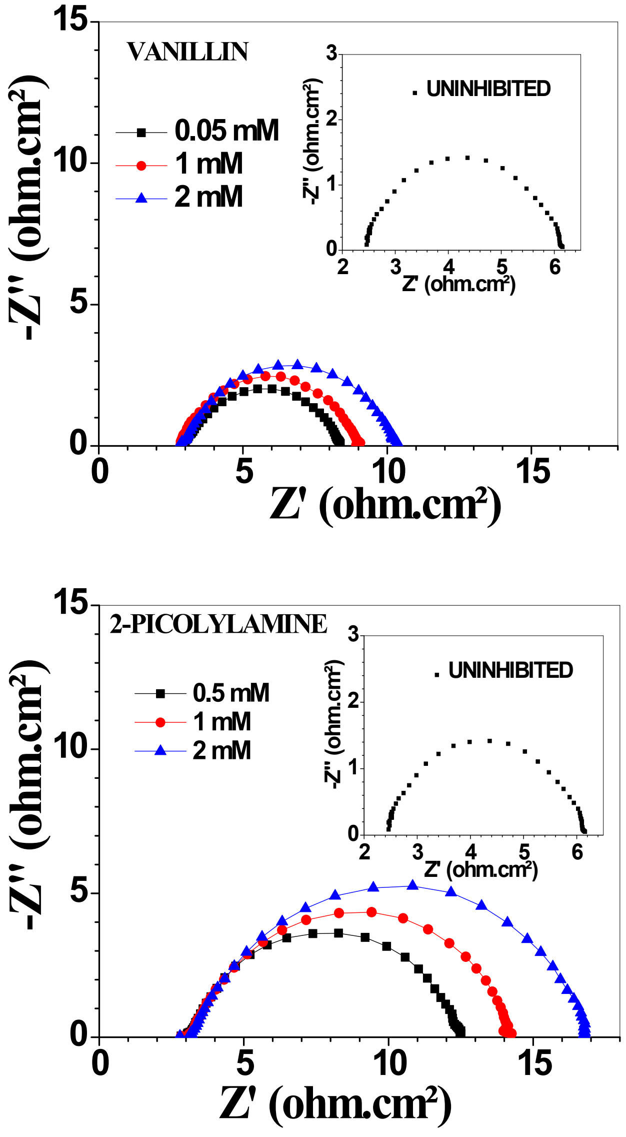

Fig. S13 Nyquist plots for mild steel in 1 M HCl in presence of vanillin (up) and 2-picolylamine (down) in 1 M HCl solution at 303K. Inset shows the corresponding plot for uninhibited sample.

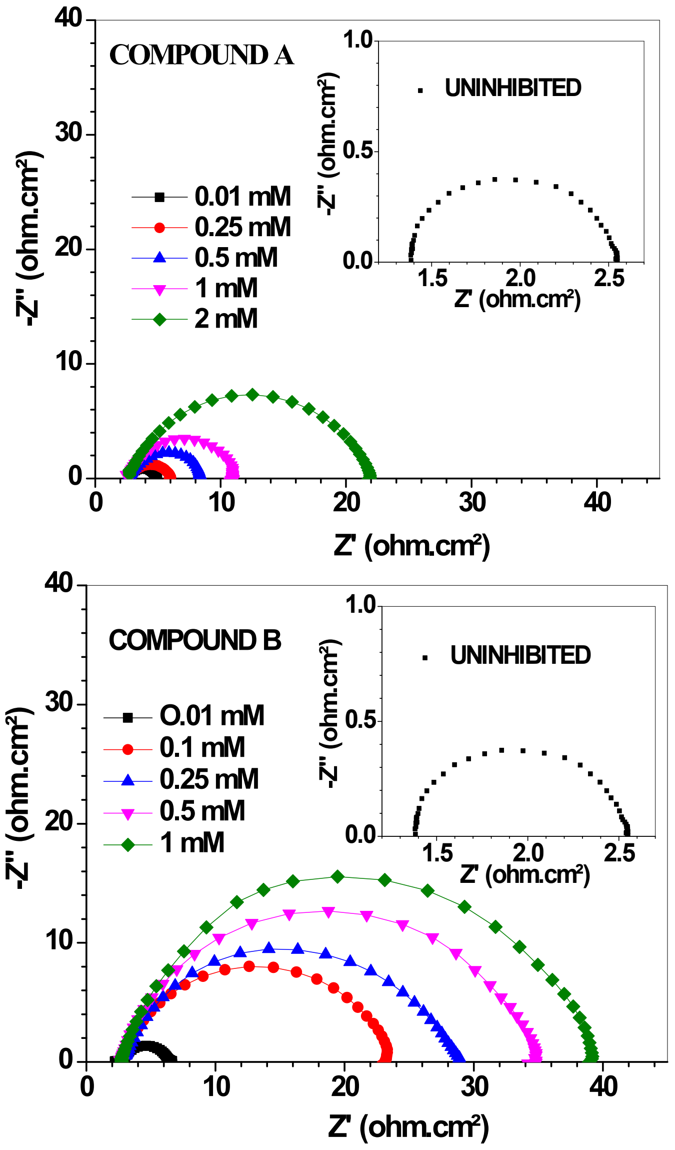

Fig. S14 Nyquist plots for mild steel in 1 M HCl in presence of compound A (up) and compound B (down) at 323K. Inset shows the corresponding plot for uninhibited sample.

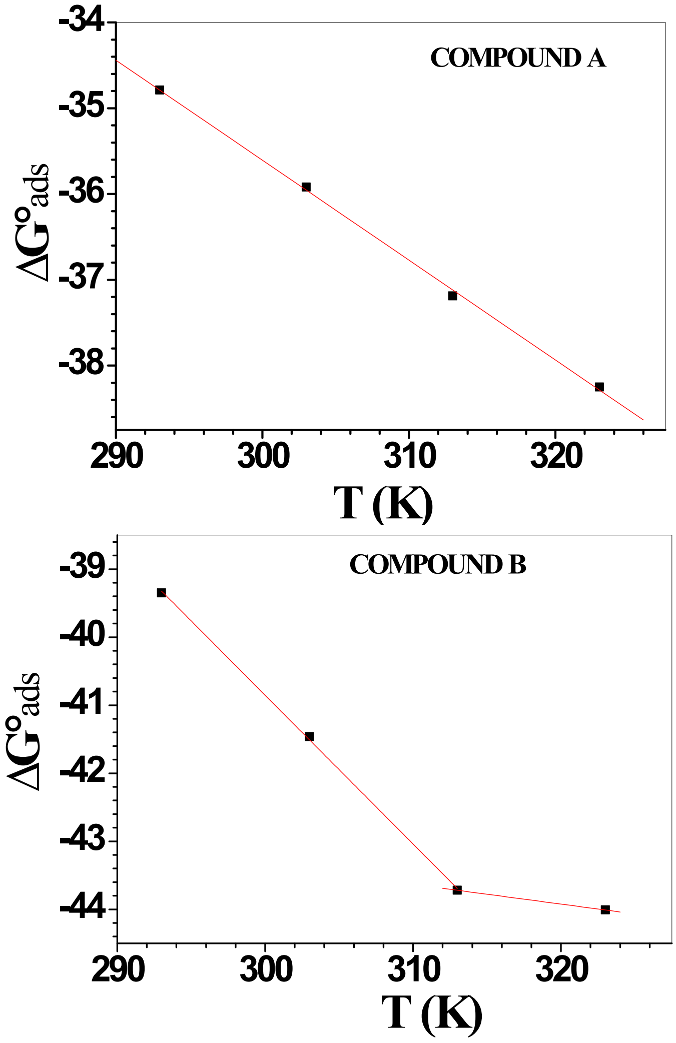

Fig. S15 Standard free energy of adsorption *vs*. Temperature plot of compound A (up) and compound B (down)

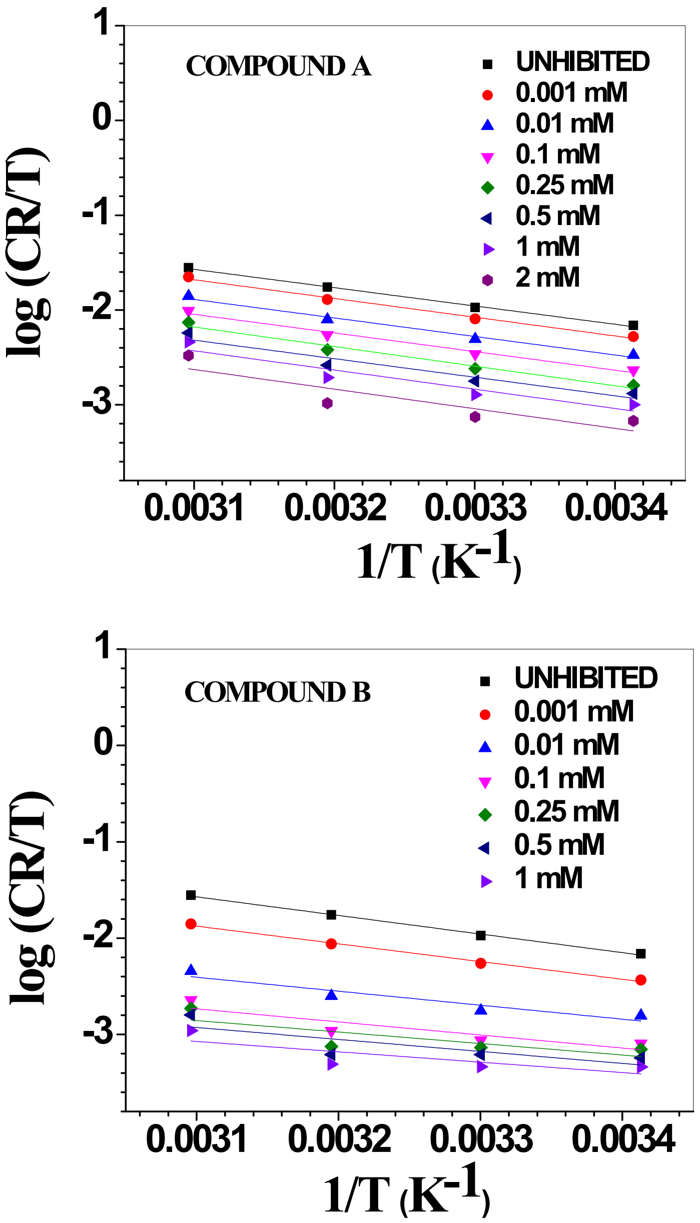

Fig. S16 Arrhenius plots for mild steel in 1 M HCl solution in absence and presence of compound A (up) and compound B (down).

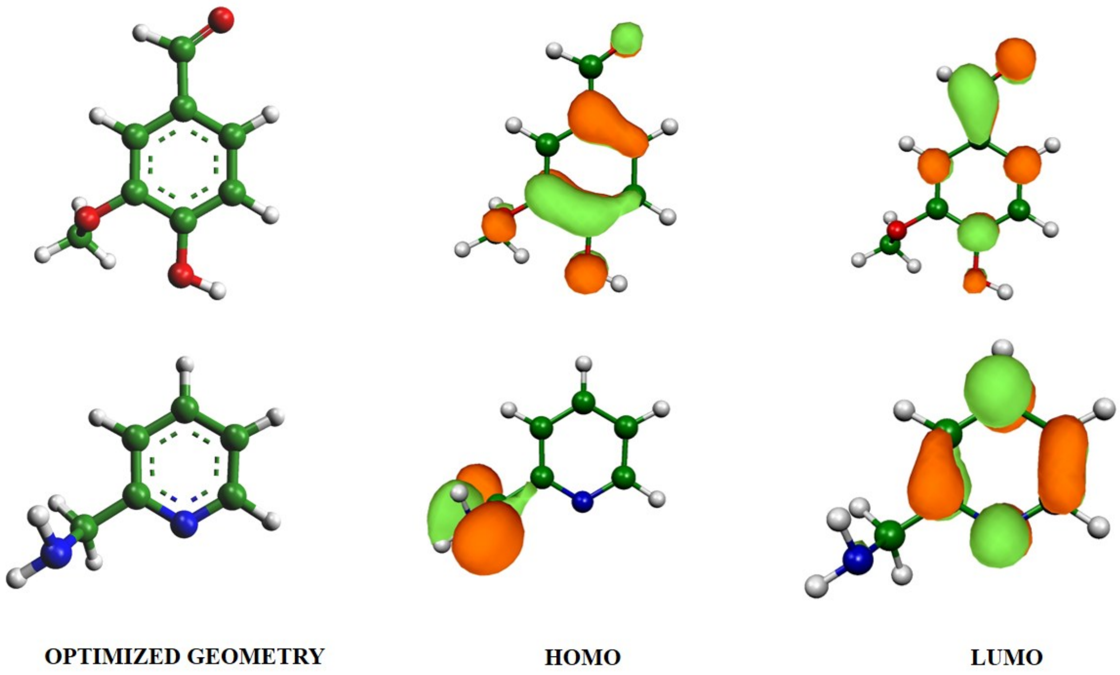

Fig. S17 Optimized geometry and electron distribution in HOMO and LUMO for vanillin (up) and 2-picolyl amine (down) as obtained from DFT study

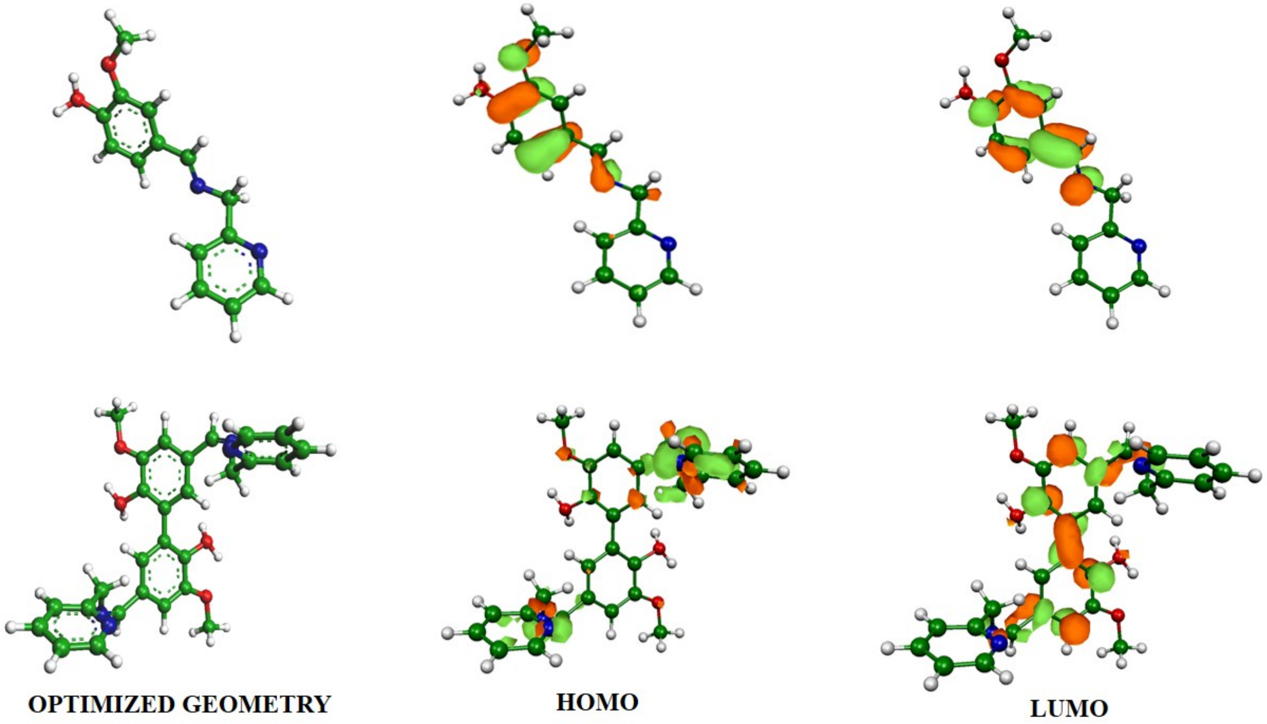

Fig. S18 Optimized geometry and electron distribution in HOMO and LUMO for compound A in monoprotonated form (up) and compound B in di-protonated form (down) as obtained from DFT study

Table S1 Potentiodynamic polarisation data for mild steel in 1 M HCl in presence of vanillin and 2-picolyl amine at 303K

**Conc. of inhibitor (mM)**	**$-E_{corr}$ (mV/SCE)**	**b_a (mVdec^{-1})**	**$-b_c$ (mVdec^{1})**	**i_{corr} (µA cm^{-2})**	**η_p%**
UNINHIBITED	521	121.4	158.7	3254	-
		vanillin			
0.001	508	105.6	133.4	2858	12.2
0.010	512	106.5	133.3	2819	13.4
0.10	507	107.7	133.1	2606	19.9
0.25	509	104.5	133.8	2337	28.2
0.50	519	107.4	135.4	2006	38.3
1.0	519	105.6	135.7	1709	47.5
2.0	512	107.1	133.4	1556	52.2
		2-picolylamine			
0.001	495	106.9	133.7	2712	16.6
0.010	506	105.8	134.1	2314	28.9
0.10	499	106.1	133.6	2133	34.5
0.25	507	106.0	134.3	1857	42.9
0.50	491	107.2	134.1	1786	45.1
1.0	499	106.6	133.5	1702	47.7
2.0	526	105.1	132.2	953	70.7

Table S2 Potentiodynamic polarisation data for mild steel in 1 M HCl in presence and absence of compound A and compound B at 323K

**Conc. of inhibitor (mM)**	**-E_{corr} (mV/SCE)**	**b_a (mVdec^{-1})**	**-b_c (mVdec^{-1})**	**i_{corr} (μA cm^{-2})**	**η_p%**
UNINHIBITED	513	116	147.6	11048	-
		Compound A			
0.001	496	104.3	128.5	8729	21.0
0.010	494	103.6	128.9	7592	31.3
0.10	512	104.4	125.3	5257	52.4
0.25	500	104.0	128.5	3944	64.3
0.50	501	104.3	127.5	3034	72.5
1.0	503	103.6	127.9	2062	81.3
2.0	518	105.3	128.5	990	91.0
		Compound B			
0.001	513	101.4	129.3	8301	24.9
0.010	514	103.3	129.9	2405	78.2
0.10	515	104.4	125.5	909	91.7
0.25	515	103.9	127.3	633	94.3
0.50	516	102.8	127.7	590	94.7
1.0	527	104.1	127.3	399	96.4

Table S3 EIS data for mild steel in 1 M HCl in presence of vanillin and 2-picolyl amine at 303K

**Conc. of inhibitor (mM)**	R_p (Ωcm^2)	Q ($\mu\Omega^{-1}s^n$ cm^{-2})	n	C_{dl} (μF cm^{-2})	η_z%	$\chi^2 \times 10^4$
UNINHIBITED	3.8	1010	0.81	273.9		4.21
			vanillin			
0.001	3.6	1680	0.80	468.5	-	2.41
0.010	3.9	1360	0.80	367.0	2.6	3.41
0.10	4.2	970	0.81	266.8	9.5	3.04
0.25	4.6	819	0.81	221.2	17.4	2.34
0.50	5.3	727	0.81	197.4	28.3	2.444
1.0	6.3	667	0.81	184.8	39.7	3.27
2.0	7.6	626	0.82	193.5	50.0	2.98
			2-picolylamine			
0.001	3.8	687	0.82	186.2	-	2.77
0.010	4.2	665	0.82	182.9	9.5	2.99
0.10	6.2	632	0.82	187.2	38.7	3.23
0.25	7.8	525	0.82	157.0	51.3	2.62
0.50	9.3	422	0.83	135.7	59.1	2.61
1.0	11.1	353	0.83	113.5	65.8	3.52
2.0	13.7	313	0.83	102.5	72.3	3.17

Table S4 EIS data for mild steel in 1 M HCl in presence and absence of Compound A and compound B at 323K

Conc. of inhibitor (mM)	R_p (Ωcm^2)	Q ($\mu\Omega^{-1}s^n$ cm^{-2})	n	C_{dl} (μF cm^{-2})	η_z%	$\chi^2\times10^4$
UNINHIBITED	1.2	1980	0.82	525.6	-	0.51
			Compound A			
0.001	1.3	3000	0.85	1127.2	7.7	0.44
0.010	1.6	2014	0.85	731.7	25.0	0.47
0.10	2.3	817	0.85	269.9	47.8	3.2
0.25	3.4	769	0.85	269.3	64.7	1.22
0.50	5.8	361	0.85	121.5	79.3	1.01
1.0	8.5	322	0.86	123.2	85.9	2.69
2.0	19.4	216	0.86	88.6	93.8	2.17
			Compound B			
0.001	1.6	1680	0.82	458.2	33.3	0.61
0.010	3.6	1280	0.83	425.3	66.7	2.81
0.10	21.0	277	0.83	96.5	94.3	.64
0.25	25.4	256	0.85	105.3	95.3	1.59
0.50	31	168	0.86	71.4	96.1	0.69
1.0	36.9	157	0.86	67.9	96.8	0.86

Table S5 Corrosion rate of mild steel in 1 M HCl in presence and absence of 1 mM compound A and compound B at different exposure times at 303K

**Exposure Time (h)**	**Inhibitor**	**Weight loss (mg)**	**Corrosion Rate (mg cm^{-2} h^{-1})**	**η_W%**
6	Uninhibited	259.8	3.207	-
	Compound A	31.3	0.386	88.0
	Compound B	11.3	0.140	95.6
24	Uninhibited	1210.3	3.735	-
	Compound A	142.8	0.441	88.2
	Compound B	49.6	0.153	95.9
48	Uninhibited	1796.7	2.773	-
	Compound A	262.3	0.405	85.4
	Compound B	116.8	0.180	93.5
72	Uninhibited	2141.5	2.203	-
	Compound A	359.8	0.370	83.2
	Compound B	192.7	0.198	91.0
96	Uninhibited	2453.7	1.893	-
	Compound A	505.5	0.390	79.4
	Compound B	282.2	0.217	88.5

Table S6. Calculated Fukui functions of the atoms present in compound A, compound B, vanillin and 2-picolyl amine respectively with their numbering of theirs atoms.

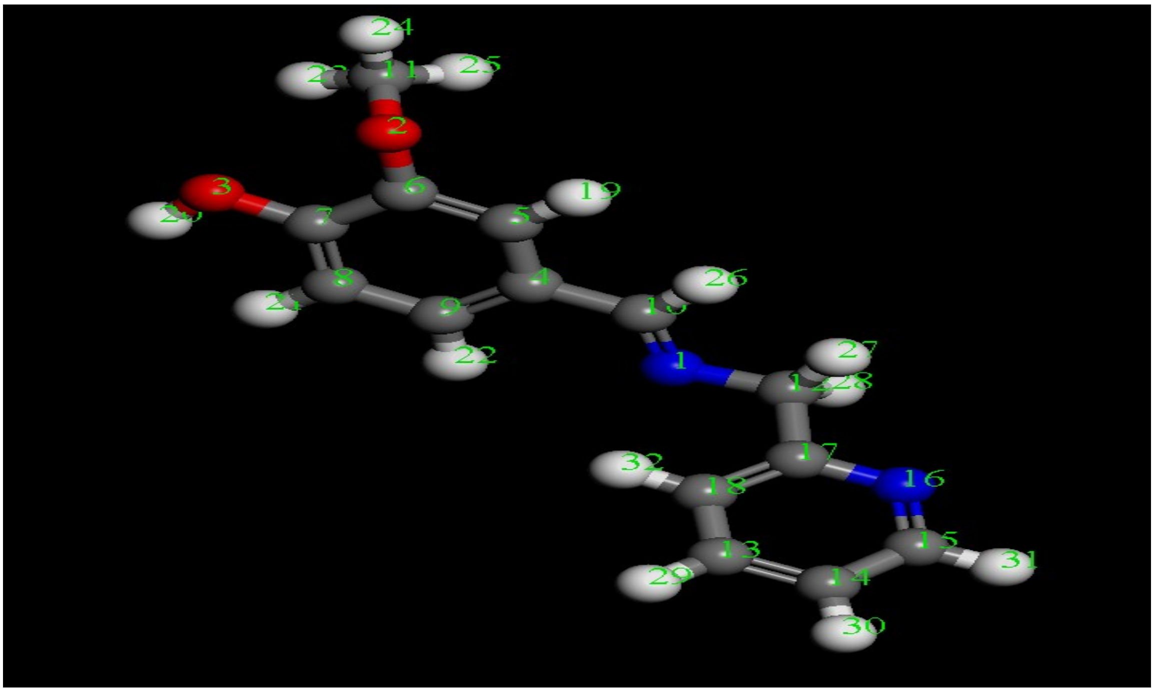

Compound A		
atom	f_k^+	f_k^-
N (1)	0.109	0.073
O (2)	0.017	0.047
O (3)	0.047	0.103
C (4)	0.053	0.085
C (5)	0.055	0.046
C (6)	0.035	0.068
C (7)	0.067	0.081
C (8)	0.042	0.057
C (9)	0.070	0.067
C (10)	0.120	0.040

C (11)	0.008	0.017
C (12)	0.023	0.018
C (13)	0.021	0.007
C (14)	0.016	0.009
C (15)	0.017	0.009
N (16)	0.022	0.015
C (17)	0.012	0.006
C (18)	0.013	0.007
H (19)	0.028	0.028
H (20)	0.019	0.036
H (21)	0.023	0.030
H (22)	0.034	0.031
H (23)	0.006	0.073
H (24)	0.007	0.047
H (25)	0.007	0.103
H (26)	0.048	0.085
H (27)	0.020	0.046
H (28)	0.028	0.068
H (29)	0.010	0.081
H (30)	0.008	0.057
H (31)	0.009	0.067
H (32)	0.010	0.040

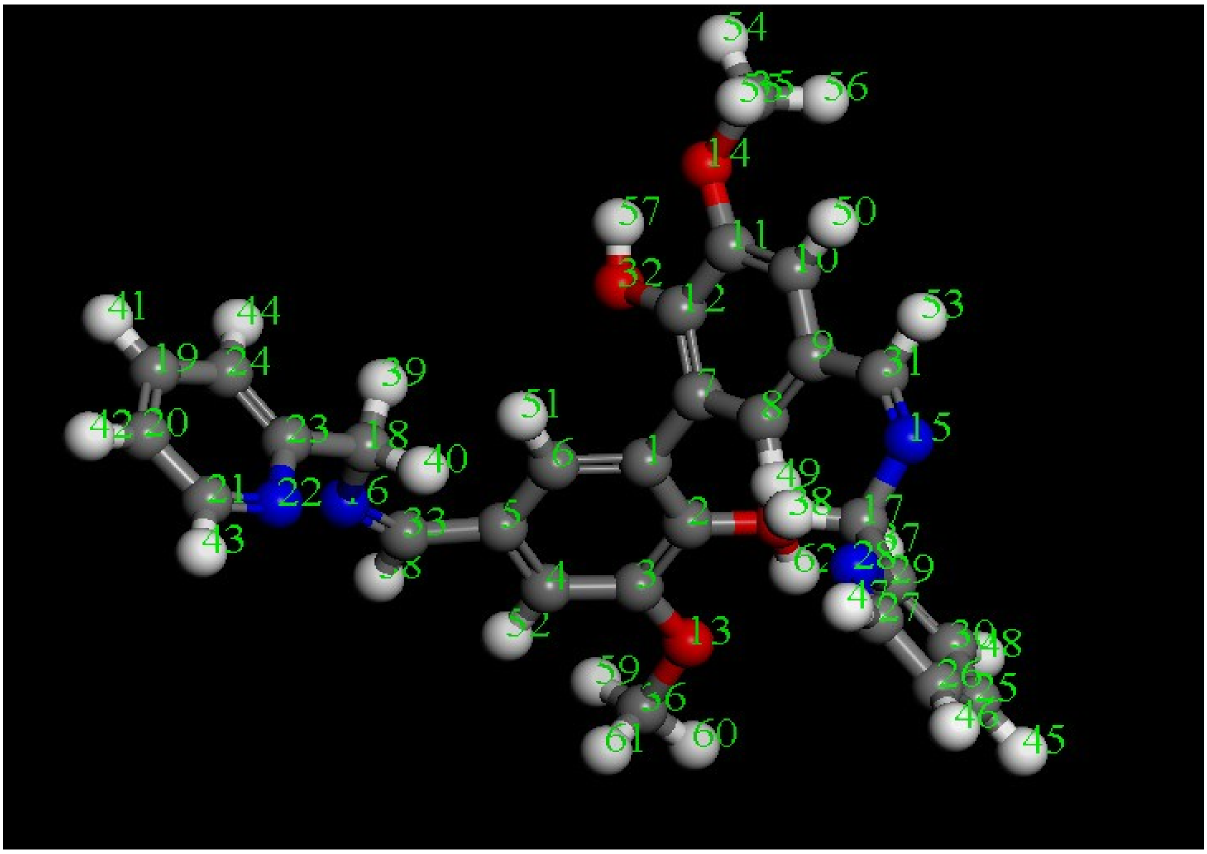

Compound B		
atom	f_k^+	f_k^-
C (1)	0.015	0.022
C (2)	0.036	0.042
C (3)	0.019	0.037
C (4)	0.033	0.028
C (5)	0.028	0.040
C (6)	0.028	0.035
C (7)	0.016	0.021
C (8)	0.028	0.035
C (9)	0.028	0.040
C (10)	0.034	0.027

C (11)	0.019	0.036
C (12)	0.037	0.041
O (13)	0.011	0.039
O (14)	0.011	0.039
N (15)	0.063	0.042
N (16)	0.062	0.043
C (17)	0.011	0.007
C (18)	0.011	0.007
C (19)	0.010	0.003
C (20)	0.008	0.003
C (21)	0.009	0.003
N (22)	0.009	0.004
C (23)	0.004	0.001
C (24)	0.008	0.002
C (25)	0.010	0.002
C (26)	0.008	0.003
C (27)	0.009	0.003
N (28)	0.010	0.004
C (29)	0.004	0.001
C (30)	0.008	0.002
C (31)	0.058	0.019
O (32)	0.027	0.051
C (33)	0.058	0.019
O (34)	0.027	0.051
C (35)	0.005	0.011

C (36)	0.005	0.011
H (37)	0.013	0.009
H (38)	0.012	0.008
H (39)	0.014	0.009
H (40)	0.011	0.008
H (41)	0.005	0.002
H (42)	0.004	0.002
H (43)	0.004	0.002
H (44)	0.005	0.002
H (45)	0.005	0.002
H (46)	0.004	0.002
H (47)	0.004	0.002
H (48)	0.005	0.002
H (49)	0.012	0.015
H (50)	0.015	0.015
H (51)	0.012	0.015
H (52)	0.015	0.015
H (53)	0.023	0.013
H (54)	0.004	0.008
H (55)	0.004	0.010
H (56)	0.004	0.010
H (57)	0.011	0.019
H (58)	0.023	0.013
H (59)	0.004	0.010
H (60)	0.004	0.008

H (61)	0.004	0.010
H (62)	0.011	0.019

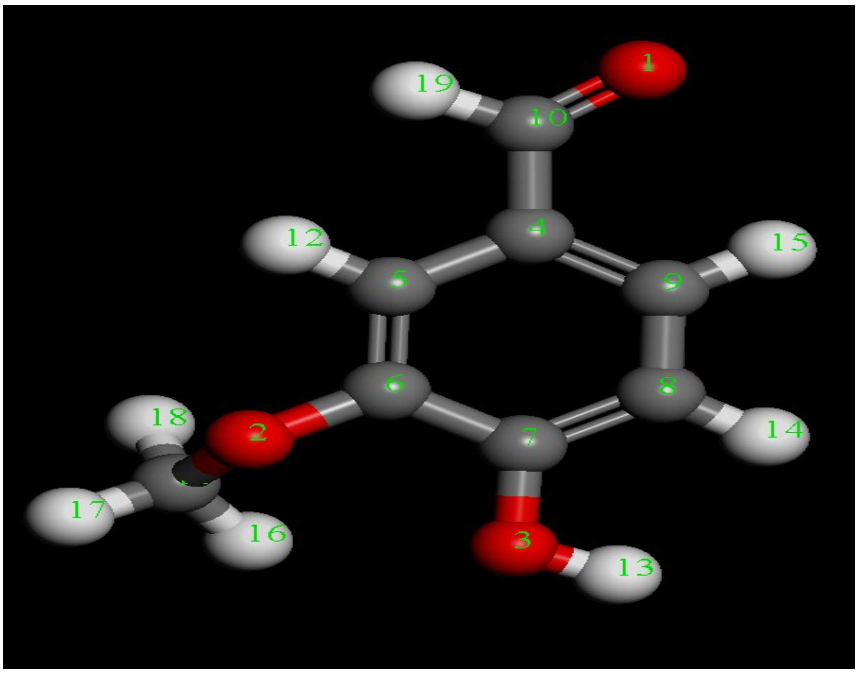

**vanilin**		
atom	f_k^+	f_k^-
O (1)	0.173	0.143
O (2)	0.019	0.064
O (3)	0.058	0.097
C (4)	0.052	0.081
C (5)	0.066	0.048
C (6)	0.037	0.074
C (7)	0.078	0.074
C (8)	0.047	0.053

C (9)	0.078	0.064
C (10)	0.175	0.062
C (11)	0.008	0.020
H (12)	0.033	0.030
H (13)	0.023	0.034
H (14)	0.026	0.030
H (15)	0.037	0.033
H (16)	0.006	0.014
H (17)	0.008	0.015
H (18)	0.008	0.018
H (19)	0.067	0.048

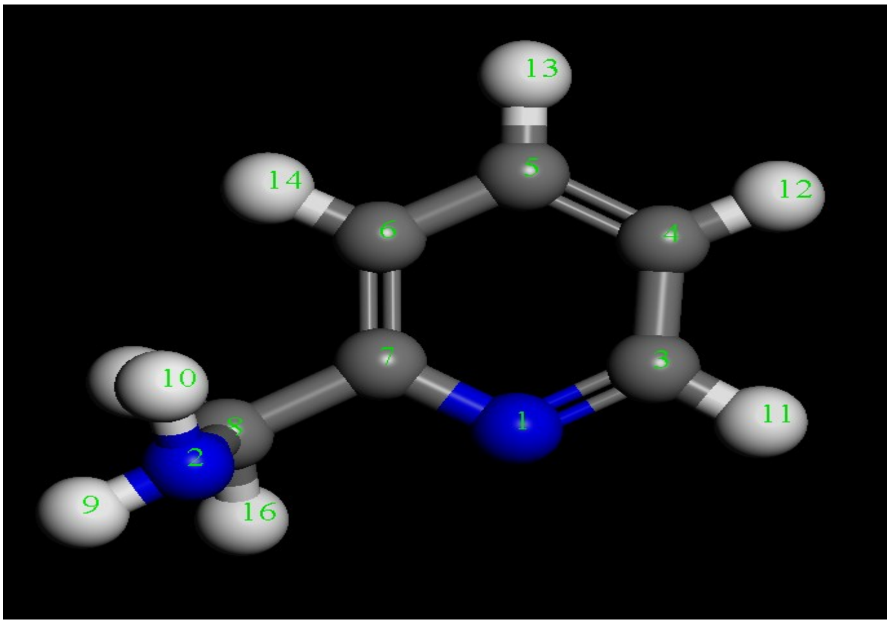

**2-picolyl amine**		
atom	f_k^+	f_k^-
N (1)	0.153	0.086

N (2)	0.020	0.328
C (3)	0.103	0.035
C (4)	0.097	0.032
C (5)	0.156	0.028
C (6)	0.082	0.028
C (7)	0.088	0.017
C (8)	0.022	0.060
H (9)	0.018	0.094
H (10)	0.013	0.091
H (11)	0.049	0.023
H (12)	0.047	0.018
H (13)	0.065	0.017
H (14)	0.043	0.019
H (15)	0.020	0.082
H (16)	0.025	0.043

Table S7 Closest distance of mild steel surface and atoms of compound A and compound B as obtained from MD simulation study

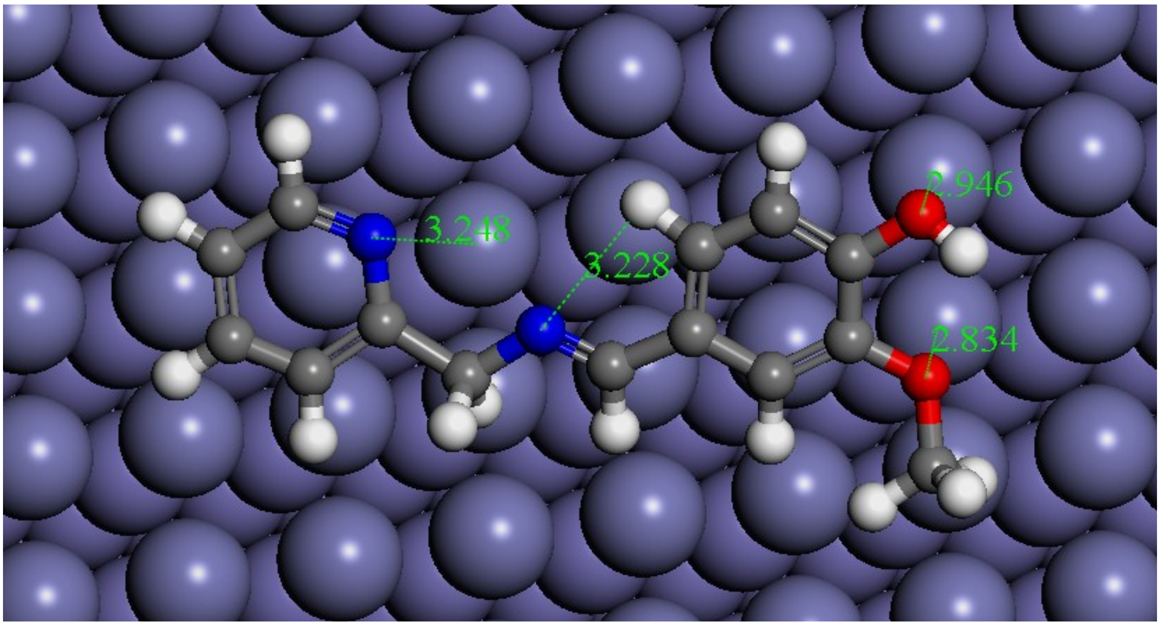

Compound A	
Fe- Atom	Closest distance
Fe- N (1)	3.228
Fe- O (2)	2.834
Fe- O (3)	2.946
Fe- N (16)	3.248

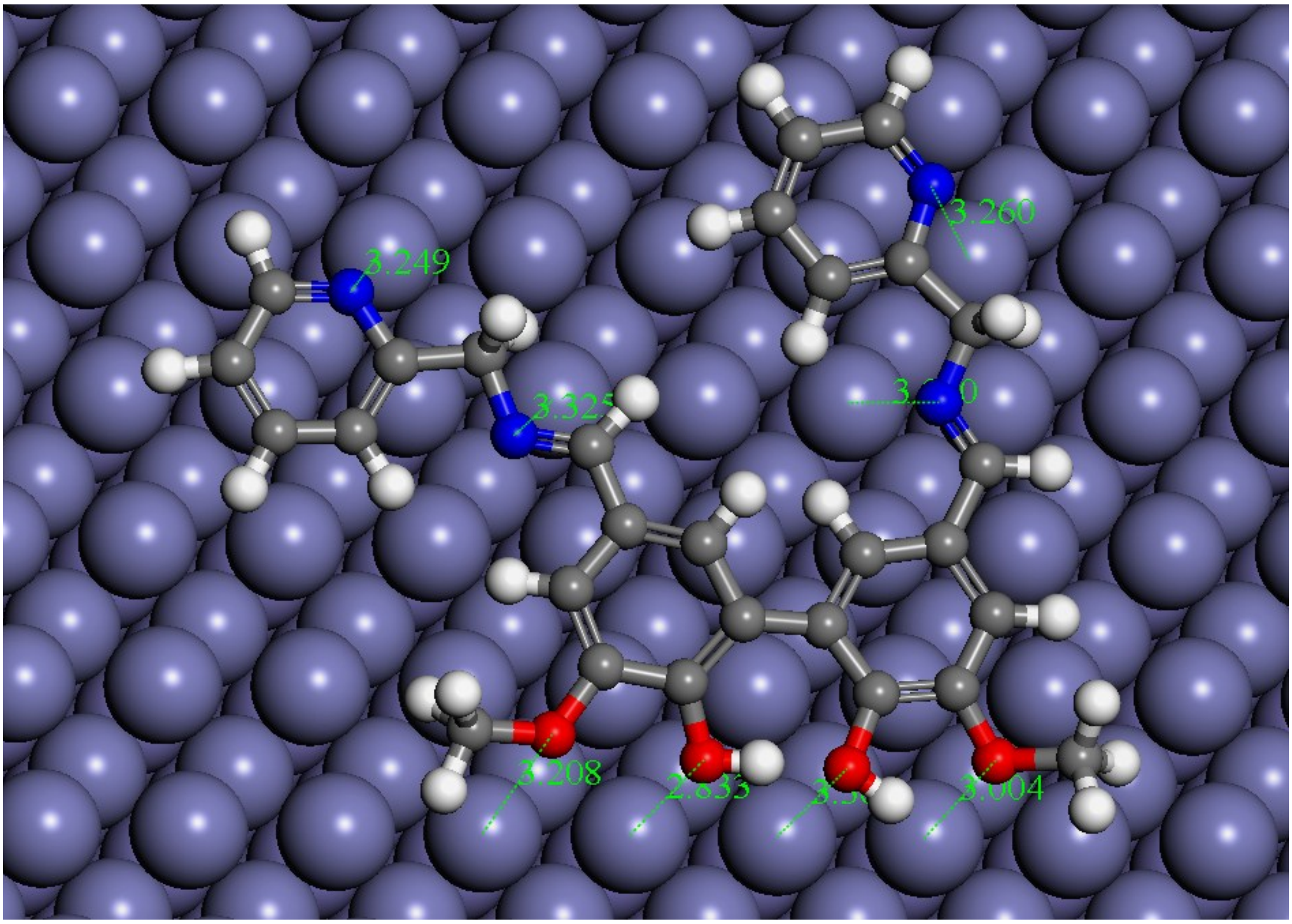

Compound B	
Fe- Atom	Closest distance
Fe- O (32)	3.369
Fe- O (14)	3.004
Fe- O (13)	3.208
Fe- O (34)	2.833
Fe- N (15)	3.080
Fe- N (28)	3.260
Fe- N (16)	3.325
Fe- N (22)	3.249
